# Autonomous circadian rhythms in the human hepatocyte regulate hepatic drug metabolism and inflammatory responses

**DOI:** 10.1126/sciadv.adm9281

**Published:** 2024-04-24

**Authors:** Sandra March, Niketa Nerurkar, Anisha Jain, Linda Andrus, Daniel Kim, Charles A. Whittaker, Edward K.W. Tan, Sabine Thiberge, Heather E. Fleming, Liliana Mancio-Silva, Charles M. Rice, Sangeeta N. Bhatia

**Affiliations:** ^1^ Institute for Medical Engineering and Science, Massachusetts Institute of Technology (MIT), Cambridge, MA 02139, USA.; ^2^ David H. Koch Institute for Integrative Cancer Research, MIT, Cambridge, MA 02139, USA.; ^3^ Broad Institute of MIT and Harvard, Cambridge, MA 02139, USA.; ^4^ Howard Hughes Medical Institute, Chevy Chase, MD 20815, USA.; ^5^ Laboratory of Virology and Infectious Disease, The Rockefeller University, NY, New York, USA.; ^6^ Institut Pasteur, Université Paris Cité, Inserm U1201, CNRS EMR9195, Unité de Biologie des Interactions Hôte-Parasite, 75015 Paris, France.; ^7^ Institut Pasteur, Université Paris Cité, Centre de Production et d’Infection des Anophèles, 75015 Paris, France.; ^8^ Wyss Institute at Harvard University, 201 Brookline Ave, Boston, MA 02215, USA.

## Abstract

Critical aspects of physiology and cell function exhibit self-sustained ~24-hour variations termed circadian rhythms. In the liver, circadian rhythms play fundamental roles in maintaining organ homeostasis. Here, we established and characterized an in vitro liver experimental system in which primary human hepatocytes display self-sustained oscillations. By generating gene expression profiles of these hepatocytes over time, we demonstrated that their transcriptional state is dynamic across 24 hours and identified a set of cycling genes with functions related to inflammation, drug metabolism, and energy homeostasis. We designed and tested a treatment protocol to minimize atorvastatin- and acetaminophen-induced hepatotoxicity. Last, we documented circadian-dependent induction of pro-inflammatory cytokines when triggered by LPS, IFN-β, or *Plasmodium* infection in human hepatocytes. Collectively, our findings emphasize that the phase of the circadian cycle has a robust impact on the efficacy and toxicity of drugs, and we provide a test bed to study the timing and magnitude of inflammatory responses over the course of infection in human liver.

## Teaser

Circadian rhythms in human liver cells control drug metabolism, inflammatory responses, and the outcome of Malaria infection.

## INTRODUCTION

The liver plays a major role in energy metabolism, xenobiotic detoxification, coordination of carbohydrate and lipid metabolism, as well as innate and adaptive immune functions against invading microorganisms. Some of these processes show daily variations in humans (*1*, *2*), and recent studies show that time of day is critical for several clinical interventions, including outcomes in response to drug treatment (*3*). This variability appears to be orchestrated by endogenous circadian clocks that mediate tissue-specific, self-sustained ~24-hour variations in gene expression termed “circadian rhythm.” In mammals, the molecular basis for these physiological rhythms is coordinated by a small group of genes called “core clock genes.” One of these genes,* brain and muscle ARNT-like 1* (*Bmal1*), serves as a master regulator of the system, acting as a transcription factor that heterodimerizes with another transcription factor, *circadian locomotor output cycles kaput* (*Clock*), to activate circadian gene expression. The BMAL1-CLOCK heterodimer acts in a series of feedback loops with other core clock genes to control 24-hour rhythmic expression of ~50% of mammalian genes, resulting in important physiological and therapeutic implications (*4*–*8*). In particular, the liver displays a large number of circadian-dependent genes and pathways, which have been shown to affect liver function in health and disease (*7*, *9*–*11*). Endogenous clocks in the liver play fundamental roles in maintaining liver homeostasis by regulating glucose and lipid metabolism (*12*, *13*). Several inflammatory responses are also circadian-regulated. Studies in mouse models and humans have shown diurnal variations in several immune-related processes, such as circadian variations in T cell subpopulations in human blood (*14*), the trafficking of monocytes to sites of inflammation in mice (*15*), and the dendritic cells to the tumor draining lymph node (*16*). Humans appear to be more susceptible to infections and more responsive to vaccinations at certain times of day (*17*–*20*), and in mice, changes in host responses to bacterial endotoxin or infection at different times of day have been documented (*21*). Furthermore, drug pharmacokinetics in the liver—including distribution, uptake, metabolism, and elimination of drug compounds—are regulated by transcription factors that are under the control of the circadian clock (*9*).

Although animal models have been instrumental for our understanding of circadian biology, the drug metabolism profile and some aspects of the innate immune response are unique in humans (*22*, *23*). Moreover, although the core clock genes are similar between species, this set of genes target and regulate the expression of core-controlled genes that tend to be species and tissue specific and typically contribute to essential tissue functions (*4*, *6*, *8*, *24*, *25*). Therefore, human in vitro systems could offer an alternative to overcome the disconnect that can arise when using animal models and would facilitate a test bed with which to explore the influence of circadian genes in different aspects of liver pathophysiology and to deploy a more efficient drug development pipeline. Despite the documented role that circadian control of human gene expression plays in several physiological processes and the abundance of cycling genes in the liver (*6*, *8*), we still lack experimental systems to enable the study of these pathways in human liver cells necessary to bridge between circadian-driven cellular processes and preclinical studies.

Here, we optimized a previously well-characterized primary human hepatocyte (PHH) in vitro system, wherein cryopreserved PHHs are organized in micropatterned islands among supportive stromal fibroblast cells, thereby stabilizing the function of liver parenchymal cells for 4 to 6 weeks. We adapted this system to enable time synchronization and observed the emergence of circadian rhythms, as confirmed by the detection of self-sustained oscillations over a period of 10 weeks under constant, unperturbed conditions. Notably, the addition of circadian oscillations prolonged the functional stability of the cultures for periods longer than 10 weeks. To assess the impact of circadian oscillations on the human hepatocyte, we generated gene expression profiles corresponding to 16 successive time points over a continuous period of two 24-hour cycles. Analyses of these data identified a set of cycling genes, including a subset of well-established core clock genes and other oscillating genes related to inflammation, drug metabolism, and energy homeostasis. Notably, knockdown experiments confirmed that many of these genetic programs were dependent on the expression of *Bmal1*. Furthermore, we observed circadian fluctuations of a set of drug-metabolizing enzymes and inflammatory genes, a finding that points to the potential to implement this platform to model drug pharmacokinetics and the circadian inflammatory response, respectively. At the functional level, we provide examples of the impact of chronopharmacotherapy by implementing a circadian-designed treatment protocol that minimizes atorvastatin- and acetaminophen-induced hepatotoxicity and by observing that infection by hepatotropic malaria-causing *Plasmodium* parasites is driven by the host’s circadian clock.

## RESULTS

### PHHs display a circadian rhythm in culture

**Fig. 1.**
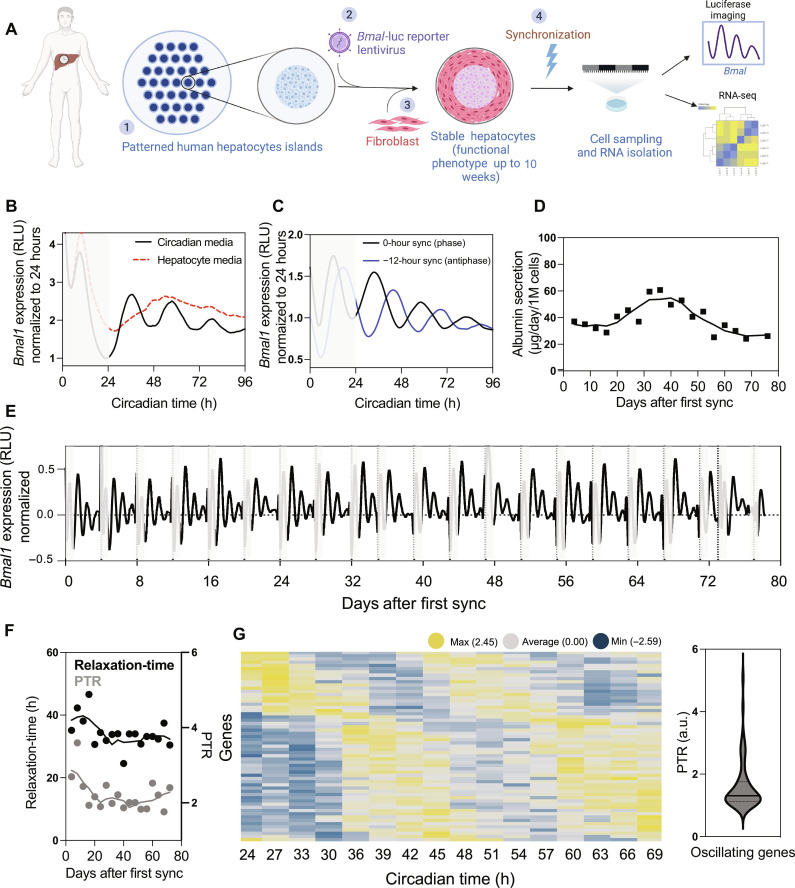
Primary human hepatocytes display a circadian rhythm in culture. (**A**) Experimental work-flow. (1) PHHs are seeded on collagen-coated plates to create hepatocyte islands. (2) Transduction with *Bmal1*-luc reporter lentiviral particles. (3) Seeding of mouse fibroblasts, 24 hours later. (4) Synchronization and monitoring of luciferase-based *Bmal1* cyclic expression. (**B**) Visualization of circadian rhythm in real time. Circadian rhythm of transduced PHH with *Bmal1*-luc reporter was monitored in real time at 20-s sampling resolution by light emission of luciferase over 4 days in free-running conditions. To synchronize the hepatocytes, the cultures were placed in specialized circadian (black) or hepatocyte media (red). For 96 hours, their circadian rhythm was monitored in real time. Circadian time is defined as hours after synchronization by media change. (**C**) Anti-phasic Bmal1 expression of PHH cultures. Synchronization of two sets of PHH cultures was performed with a circadian medium change 12 hours apart, allowing them to free-run under constant conditions for 96 hours. (**D**) Daily albumin secretion in PHH over 10-week period. (**E**) Circadian rhythm of *Bmal1*-luc reporter–transduced PHH was observed over a period of 10 weeks. Between each 96-hour monitoring of the circadian rhythms, a new synchronization of the cultures was triggered by circadian media exchange. (**F**) Hepatocytes show stable circadian relaxation-time and peak-to-trough ratio (PTR) of *Bmal1* gene expression over a 10-week period. (**G**) Transcriptomic analysis of oscillating transcripts. Heatmap representation of oscillating transcripts ordered by the time of the oscillation (columns), during a period of 48 hours. Each vertical column represents a time point (3-hour resolution). Each row is a cycling transcript, colored based on the expression intensities, low (blue) and high (yellow) (BHQ < 0.2). Expression values are mean-normalized for each gene and are ordered by peak time of expression (left). PTR for rhythmic genes (right). RLU, relative light unit; a.u., arbitrary unit.

To assess whether micropatterned cocultures (MPCCs) of PHHs are capable of displaying a circadian rhythm, we monitored Bmal1 activity in real time using a lentiviral reporter, which expresses destabilized luciferase (dluc) under control of a *Bmal1* promoter (*26*). PHH were seeded and allowed to settle onto a patterned collagen type I–coated surface, creating (500 μm in diameter) hepatocyte islands (*27*). The hepatocytes were then transduced with *Bmal1*-luc lentiviral particles as described in Materials and Methods. Following an overnight incubation, cultures were extensively washed and mouse 3T3-J2 fibroblasts were seeded and allowed to bind in the intervening space between hepatocytes islands to stabilize the functional phenotype of the PHH (*28*). Following a week of stabilization, cultures were first attempted to be synchronized by media exchange using standard hepatocyte medium and the luciferase activity was subsequently measured for 96 hours (Fig. 1A). Although beneficial in maintaining hepatocyte health (fig. S1D), the use of standard hepatocyte media was not conducive to circadian expression of the *Bmal1* reporter (Fig. 1B, red line). Upon testing of several media formulations and regimens, we found that the optimized hepatocyte medium (“circadian medium”) was necessary to visualize the cyclic expression of *Bmal1* over a 72- to 96-hour period (Fig. 1B, black line, and fig. S1, A and B). These conditions also sustained albumin levels and cytochrome P450 3A4 (CYP3A4) induction activity (a marker of a healthy hepatic functional phenotype) for up to a 10-week period (Fig. 1D and fig. S1C). This culture system was implemented and validated in two other formats (24- and 384-well plates) (fig. S1H).

Circadian behavior was confirmed in cryopreserved PHH isolated from 16 individual patient donors that range across ages and genders. The in vitro rhythm period length (the time it takes for one oscillation) varied among hepatocytes from different individuals (fig. S1, E and F). Detection of circadian rhythms was observed over a period of 10 weeks, allowing for longitudinal experiments (Fig. 1E). The amplitude of the oscillations decreased over time during each cycle, but the initial cycling pattern was restored for at least 96 hours of monitoring after each synchronization event (Fig. 1E).

The decrease in the amplitude of oscillations over time was quantitatively characterized as a characteristic exponential decay time, here referred to as “relaxation-time.” The relaxation-time values were calculated by performing discrete Fourier transform (DFT) on *Bmal1* expression levels, fitting the squared magnitude of the DFT to the expected form of a Lorentzian function, and calculating the inverse of the full width at half maximum of the Lorenztian (fig. S3A). The relaxation-time values and the peak-to-trough ratio (PTR), representing the fold difference between highest and lowest level of expression in periodic data, remained stable throughout 80 days of culture (Fig. 1F).

When two sets of hepatocyte cultures were synchronized 12 hours apart via a circadian media exchange and subsequently observed without additional perturbation (termed “free-run conditions”), these cultures showed oppositely phased rhythms known as an “antiphase” relationship for 96 hours (Fig. 1C). This antiphase rhythm was consistent in cultures of three independent PHH donors (fig. S1F). Moreover, antiphasic oscillations in the expression of *Bmal1* and *Per2* mRNA were also observed by quantitative reverse transcription polymerase chain reaction (qRT-PCR) (fig. S1G). These findings document circadian oscillations in free-running and entrainable conditions in PHH MPCC cultures, two of the hallmark properties of circadian rhythms (*29*).

### Transcriptomic analyses of synchronized PHHs

To investigate the transcripts driven by the autonomous clock in PHHs and the possible role of circadian oscillations in liver biology, we performed transcriptomic analyses in synchronized PHH MPCCs using RNA sequencing (RNA-seq). To this end, cultures were synchronized by media exchange, as described above. Twenty-four hours after synchronization, samples were collected every 3 hours across two consecutive days in independent technical triplicates, and the data were analyzed to quantify gene expression changes over time.

Gene expression data were analyzed for rhythmic oscillations using the JTK_CYCLE [Jonckheere-Terpstra-Kendall (JTK) cycle] algorithm to detect 24-hour oscillations in transcript abundance (*30*). “Circadian oscillating gene” was defined as any gene identified as oscillating with a 24-hour period by JTK_CYCLE and passing the *q* value (Benjamini-Hochberg) < 0.2 and an amplitude > 0.1 cutoff. In addition, quantitative demonstration of circadian oscillations was performed via Fourier analysis (fig. S3A), revealing a predominant contribution from an oscillatory signal with period near 24 hours (*31*, *32*).

The global analysis of results over two consecutive days using the JTK_CYCLE algorithm identified 59 oscillating genes that met a BHQ (Benjamini-Hochberg procedure) of <0.2 (*8*, *33*), and by increasing the stringency of the cutoff, 38 genes met a BHQ of <0.1 and 24 at BHQ < 0.05 (Fig. 1G and fig. S2A, top). When we focused the analysis on just the first 24 hours, we detected 388 oscillating genes at BHQ <0.2, and this collection of genes was reduced to 156 or 86 at the more stringent BHQ values of <0.1 or <0.05, respectively (fig. S2A, bottom). We identified 11 core clock genes with robust oscillation based on their BHQ of close to 0.05 (fig. S4A), and the relative peak times of each are shown in fig. S4B (far right). This conserved group of core clock genes includes *BMAL1*, *CRY2*, *PER2*, *NPAS2*, *NR1D1*, *NR1D2*, *TEF*, *HLF*, *CIART*, *PER2*, and *NFIL3*, and each showed phase correlation with other core clock genes, as previously described (*6*). *Bmal1* and *nfil3* peaked approximately 12 hours out of phase with *per2* and *nr1d1*, respectively (fig. S4B, left and middle panels). Oscillating genes displayed a PTR with a mean of 1.6. This value did not change significantly with inclusion of genes from a higher BHQ cutoff (fig. S4C). For gene expression levels of the oscillating genes identified, the relaxation-time values were calculated as previously described above (fig. S3B).

An additional analysis of the results was performed with a second algorithm, dryR (differential rhythmicit Y analysis in R). For dryR, we used the thresholds adj ≥ 0.05 and amp ≥ 0.1. DryR identified 1067 and 79 oscillating genes at 24 or 48 hours, respectively. An extensive overlap with the two algorithms was observed (fig. S2B).

To identify biological processes represented by these oscillating genes, we searched for any overlap between our list of 388 genes and the MSigDB (Molecular Signatures Database) hallmark gene sets (*34*). Hallmark gene sets that overlap with oscillating genes capture different biological categories—six of the gene sets were related to metabolic processes, including xenobiotic, fatty acid, bile acid and heme metabolism, cholesterol homeostasis, and glycolysis. Other gene sets were related to proliferation and immune responses, including gene sets involved in inflammatory and interferon (IFN) responses (fig. S2A, far right). To complement this analysis, we examined the overlap between our identified gene set and the KEGG (Kyoto Encyclopedia of Genes and Genome)/REACTOME canonical pathway collection of MSigDB. The top 10 pathways identified were mainly related with glucose, lipid, retinoid, and xenobiotics metabolism (fig. S4D). We identified rate-limiting enzymes of gluconeogenesis, such as *PCK1* (phosphoenolpyruvate carboxykinase 1) and *G6PC* (glucose-6-phosphatase alpha), the peroxisome proliferator activated receptor delta (*PPARD*) that regulates fatty acid uptake, transport, and β-oxidation playing a key role controlling lipogenesis. Other key enzymes included the alcohol dehydrogenases (*ADH1A*, *ADH1B*, and *ADH4*), and retinol dehydrogenase 5 (*RDH5*) that regulates the biosynthetic pathway for generating all-trans retinoic acid, the major biologically active retinoid in vivo. Overall, our data indicate that many genes involved in the regulation of critical metabolic functions and inflammatory processes exhibit circadian oscillations in cultured PHHs.

### Disruption of the clock in PHHs

Circadian dysfunction has been shown to induce dysregulation of liver gene expression and metabolic and immune disruption in mice (*35*, *36*). Thus, we next sought to investigate if similar events are replicated at the gene expression level when the circadian clock is disrupted in MPCC-cultured PHHs.

**Fig. 2.**
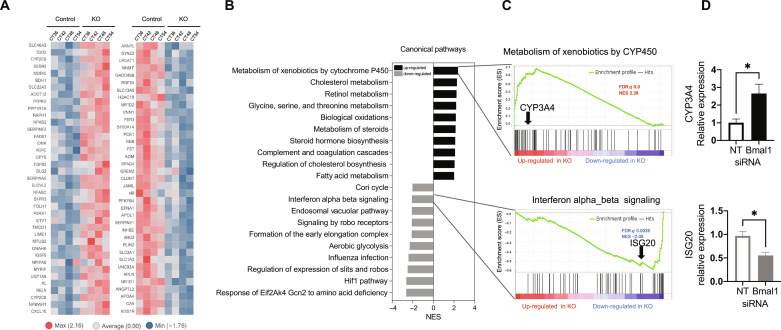
Loss of Bmal1 expression disrupts expression of genes involved in inflammatory signaling and drug metabolism in PHH. (**A**) Unclustered heatmaps of genes differentially expressed (logFC of 1, adjusted *P* of 0.05 cutoffs) in Bmal1-siRNA–treated [knockout (KO)] versus those treated with a nontargeting siRNA construct (NT). CT, circadian time in hours. (**B**) Canonical pathways GSEA. Top 10 statistically differentially expressed gene sets that were up- (black) or down-regulated (gray) in *Bmal1* knockdown cultures from MSigDB. Analysis of the CT24 time point. (**C**) Metabolism of xenobiotics by CYP450 (top) and IFN-α or IFN-β signaling (bottom) enrichment profiles. Arrows indicate the localization of *CYP3A4* and *ISG20* in the respective gene sets. (**D**) qRT-PCR validation. Up-regulation of *CYP3A4* (top) (black) and down-regulation of *ISG20* (bottom) (gray) are observed in *Bmal1* knockdown cultures. Analysis performed at CT48. FDR. false discovery rate; NES, normalized enrichment score. *P < 0.05.

To this end, we silenced the expression of *Bmal1* in human hepatocytes using small interfering RNA (siRNA) and performed transcriptomic analyses in synchronized PHH. To ensure that *Bmal1* silencing occurred solely in hepatocytes, siRNA was added to hepatocytes 6-hours after seeding (*37*). Twenty hours later, cultures were washed and fibroblasts were subsequently added. After synchronization, we collected samples 6 hours apart [at circadian time (CT) 36, CT42, CT48, and CT54]. The silencing efficiency of *Bmal1* was confirmed by qRT-PCR, and samples from each time point were subjected to RNA-seq. Substantial reduction in *Bmal1* mRNA was observed at CT42, CT48, and CT54 when siRNA-treated samples were compared to control (nontargeted) siRNA-treated PHHs (fig. S5A). For the RNA-seq datasets, differential expression analysis was performed to compare *Bmal1-*silenced and nontargeted siRNA samples at each time point, and significantly dysregulated genes were defined as those having an absolute logFC ≥ 1 and an adjusted *P* value ≤ 0.05. To investigate these changes at the gene level, replicates for each time point were averaged and these summarized values were standardized, so that each row mean was 0 and variance was 1. These data were then used to plot lists of up- and down-regulated genes at each time point, relative to the expression at CT48 (Fig. 2A). Preranked gene set enrichment analysis (GSEA) was run for the CT48 time point using the MSigDB c2cp canonical pathway collection (Fig. 2B) and the Hallmark collection (fig. S5B), leading to the detection of several dysregulated signaling pathways. Substantial changes included the upregulation of pathways related to the metabolism of xenobiotics by CYP450 enzymes (Fig. 2B). The leading edge, or far left side, of this waterfall plot included genes that belong to the core drug absorption, distribution, metabolism, and excretion (ADME) gene set, which represent the most important genes directly involved in drug metabolism and clearance (Fig. 2C, top, and fig. S5C, left). *CYP3A4* was one of the differentially expressed genes that we further validated by qRT-PCR (Fig. 2D, top). We also identified the regulation of pathways involved in inflammatory responses and IFN signaling (Fig. 2B and fig. S5B). In particular, also among the leading edge genes were those involved in cytokine and chemokine signaling (fig. S5B) and several IFN-stimulated genes (ISGs) (Fig. 2C, bottom, and fig. S5C, right). The decreased expression of one of them, *ISG20*, was validated by qRT-PCR (Fig. 2D, bottom). Overall, these observations demonstrated that *Bmal1* controls circadian expression of genes involved in inflammatory signaling and drug metabolism in cultured PHHs, and thus, we focused on these processes to further study their potential biological implications.

### Assessment of circadian drug metabolism and CYP3A4 hepatotoxicity

**Fig. 3.**
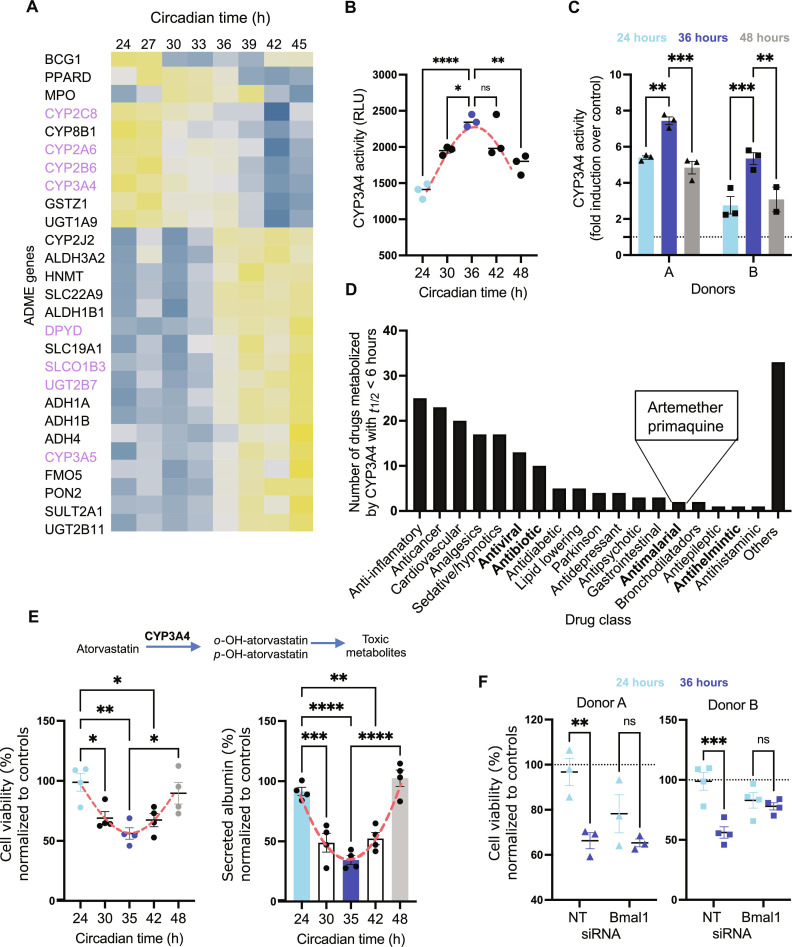
PHH exhibit circadian-dependent patterns of drug metabolism. (**A**) Transcriptomic analysis of oscillating transcripts involved in drug metabolism. Phase sorted heat map of cycling ADME genes over a 24-hour period. Each vertical column represents a time point (3-hour resolution). Each row is a cycling transcript, colored based on the expression intensities, from low (blue) to high (yellow) (BHQ < 0.2). Expression values are mean-normalized for each gene and are ordered by the peak of expression. (**B**) CYP3A4 luminogenic activity assay. An activity-based luminogenic assay was used to determine CYP3A4 enzymatic activity in synchronized hepatocytes every 6 hours, over a 24-hour period. (**C**) Impact of rifampin induction on CYP3A4 activity. Synchronized cultures at CT24, CT36, and CT48 were dosed with rifampin (10 μM), and 24 hours later, CYP3A4 activity was measured via luminogenic assay. (**D**) Putative candidates for chronotherapy. Drugs metabolized by CYP3A4 with *t*_½_ < 6 hours. (**E**) Atorvastatin hepatotoxicity mediated by CYP3A4. Schematic showing the metabolism of atorvastatin by CYP3A4 and its by-products (top). Adenosine 5′-triphosphate (ATP)–based real-time viability assay to determine CYP3A4-metabolized toxicity of atorvastatin in PHH cultures synchronized 5 to 7 hours apart (CT24 versus CT30 versus CT35 versus CT42 versus CT48). Atorvastatin was added (200 μM) and viability was monitored for 2 hours (bottom left). Albumin levels measured 12 hours after dosing with atorvastatin (bottom right). CT24, CT30, CT35, CT42, and CT48 are defined, respectively as, 24, 30, 35, 42, and 48 hours after synchronization by media change. (**F**) Circadian-dependent Atorvastatin hepatoxicity in Bmal1-silenced hepatocytes. Atorvastatin was added at 200 μM, and real-time viability was measured for 2 hours in two independent PHH donors. Data are mean ± SEM. *n* = 3 or *n* = 4 independent wells. Statistical significance was determined using a two-way analysis of variance (ANOVA) with post hoc pairwise comparisons. ****P < 0.0001, ***P < 0.001,**P < 0.01, and *P < 0.05.

Circadian-mediated expression of ADME genes can affect the pharmacokinetics of a drug, which could influence its efficacy, toxicity, or therapeutic index (*3*, *9*). Now, 299 genes that encode phase I and II drug-metabolizing enzymes, transporters, and modifiers are designated as ADME genes by the PharmaADME Consortium. Therefore, we examined the expression of this gene set in the global circadian transcriptome previously described in Fig. 1 and identified 27 transcripts (9% of the ADME gene set) that oscillate in a circadian pattern in our cultures (Fig. 3A and fig. S6A). Of these genes, eight (*CYP2C8*, *CYP2A6*, *CYP2B6*, *CYP3A4*, *DPYD*, *SLCO1B3*, *UGT2B7*, and *CYP3A5*) belonged to the Core ADME set comprising 32 genes, which represents the most important genes directly involved in drug metabolism and clearance. The other 19 genes belonged to the extended ADME gene set that represents other genes related to drug metabolism and clearance (fig. S6C). Most of the cycling genes belonged to the phase 1 metabolism group (enzymes responsible for redox reactions to generate metabolically active polar groups) including six genes of the core ADME gene set (fig. S6, A and C). Consistent with previous studies, we observed two waves of gene expression whose relative circadian peaks are shown as polar histograms in fig. S6B. Given their importance in clinically relevant drug metabolism, further biochemical and functional validation of cytochromes CYP3A4 and CYP2B6, and the transporter, SLCO1B3 was performed. The oscillating expression of these three genes was confirmed by qRT-PCR and at the protein level by Western blotting (fig. S6D). CYP3A4 and CYP2B6 had the highest peak of protein expression at CT32, and SLCO1B3 peaked at CT40.

Having established the circadian expression of CYP3A4 at both the mRNA and protein levels and given the relevance of CYP3A4-mediated drug metabolism, we next assessed the circadian oscillation of CYP3A4 enzymatic activity. To this end, we used a luminogenic CYP3A4 activity-based assay that specifically measures cellular CYP3A4 activity by converting a luminogenic substrate into luciferin, which is then released into the culture media and further converted into light in the presence of luciferase. Twenty-four hours after synchronization, CYP3A4 enzymatic activity was measured every 6 hours (CT24 to CT48). As seen in Fig. 3B, these experiments documented circadian oscillation of CYP3A4 enzymatic activity in PHH, which peaked at CT36. These results suggested that the pharmacokinetics of a given drug may differ depending on the time of the day it is delivered. Therefore, next, we sought to identify drugs metabolized by CYP3A4 with a half-life less than 6 hours. Following this criteria, we identified 189 drugs that could potentially benefit from chronotherapy including for the anti-malarial drugs artemether and primaquine (Fig. 3D and table S1). Next, we functionally evaluated circadian-dependent induction of CYP3A4 using rifampin, a well-known inducer of CYP3A4. Induction of CYP3A4 expression is often implicated in clinically relevant drug-drug interactions (DDIs) because metabolism catalyzed by this enzyme is the dominant route of elimination for many drugs (*38*). Twenty-four hours after synchronization, cultures were dosed with 2.5 μM rifampin at CT24, CT36, or CT48, and enzymatic activity of CYP3A4 was measured 24 hours later. The induction of CYP3A4 activity in two independent PHH donors treated with rifampin showed a circadian pattern. In that activity was higher when rifampin was added at CT36 versus CT24 or CT48, with a fold difference of 1.6 (Fig. 3D and fig. S6E).

Next, we performed a proof-of-concept toxicity analysis using two known hepatotoxicants, atorvastatin (ATOV) or acetaminophen [*N*-acetyl-*p*-aminophenol (APAP)]. ATOV is primarily used as a lipid-lowering agent, owing to its capacity to inhibit the 3-hydroxy-3-methyl-glutaryl-coenzyme A reductase, an enzyme found in liver tissue that plays a key role in the production of cholesterol. ATOV is metabolized by CYP3A4 to form ortho- and para-hydroxylated metabolites (Fig. 3E). While the mechanism underlying atorvastatin-induced hepatotoxicity has not been well established, it likely involves increased formation of reactive oxidative species during metabolism (*39*). To evaluate circadian-dependent hepatoxicity in PHHs, we assessed cell viability in real time using a luminescence adenosine 5′-triphosphate–based assay. Synchronized PHHs were treated with scaling doses of ATOV for 120 min [Fig. 3E and fig. S6, F (left) and G (top)]. We observed higher hepatoxicity in the PHH treated at CT35 compared to the cultures treated at CT24 or CT48 (Fig. 3E, left). This toxicity correlated with the higher levels of CYP3A4 activity observed at CT36 in Fig. 3B and which was confirmed 30 min before the cell viability assay was performed. Using a different readout, we detected lower levels of albumin 12 hours after dosing with ATOV at CT35 versus CT24 and CT48 (Fig. 3E, right).

Next, we silenced the expression of *Bmal1* in the human hepatocytes using siRNA as described above and evaluated the circadian-dependent hepatoxicity to ATOV. Using two independent PHH donors, we showed an ablation of the circadian hepatoxicity to ATOV (Fig. 3F).

Together, our observations confirmed the circadian dependence of CYP3A4-mediated hepatoxicity.

Next, we tested whether APAP hepatotoxicity would also display a circadian pattern. APAP is a widely-used analgesic that can cause acute hepatic necrosis when administered at high doses due to the formation of a toxic metabolite (*N*-acetyl-*p*-benzoquinone imine) produced when CYP3A4, CYP2E1, CYP1A2, or CYP2A6 acts on the parent drug (fig. S3F). PHHs were treated with scaling doses of APAP, and their viability was measured in real time, as above [fig. S6, F (right) and G (bottom)]. After APAP incubation, we observed higher hepatoxicity in the PHH treated at CT36 compared to the cultures treated at CT24 (fig. S3F). As observed after ATOV treatment, APAP hepatocyte toxicity correlated with the higher levels of CYP3A4 activity at CT36 (Fig. 3B). Overall, these experiments establish the feasibility of developing treatment protocols to minimize drug hepatotoxicity and adverse effects and the capacity to use the MPCC platform to test these protocols in PHH.

### Circadian control of the hepatocyte inflammatory response and infection

Further analyses of our transcriptomic data led to the identification of inflammatory genes that exhibit circadian-regulated expression patterns. This subset of genes includes several categories: (i) genes involved in the IFN response, ISGs such as *CXCL10*, *USP18*, *PARP14*, *GBP2*, *PARP9*, *MT2A*, *TNFSF10*, and *IFIT1*; (ii) genes related to the interleukin-2 (IL-2) signal transducers and activators of transcription 5 signaling pathways, such *NFIL3*, *TGM2*, *ALCAM*, *CXCL10*, *TNFSF10*, *APLP1*, *ENPP1*, and *SLC39A8*; (iii) oscillating transcripts related to the inflammatory response, such *RAF1*, *ROS1*, *IFIT1*, *SLC7A2*, *CXCL10*, and *TNFSF10*; and (iv) genes involved in innate immunity, such *CD14*, *CRP*, and *HEPCIDIN* (fig. S7A). For most of these examples, the peak oscillating gene expression was observed between 18 and 20 hours after the peak of *Bmal1* expression (fig. S7B), suggesting a tightly coordinated response of inflammatory gene expression.

**Fig. 4.**
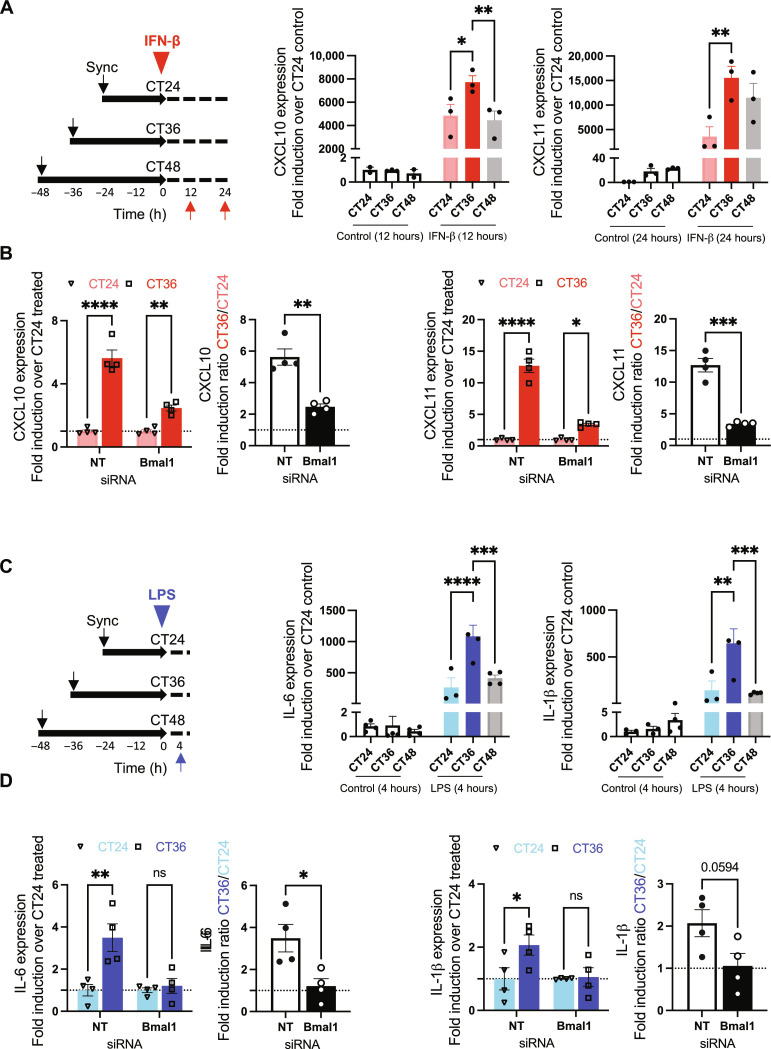
Circadian control of the hepatocyte inflammatory response. (**A**) Circadian-dependent inflammatory response to an immune challenge mimicked by IFN-β exposure. Schematic of induction via immune stimuli challenge (left). Expression of inflammatory cytokines was measured by qPCR. mRNA was isolated from synchronized hepatocyte cultures harvested at 12 and 24 hours after IFN-β treatment (1000 U/ml) at either CT24 (light red), CT36 (dark red), or CT48 (gray). Levels of cytokine mRNA were quantified [relative to glyceraldehyde-3-phosphate dehydrogenase (GAPDH)] and presented in relation to expression levels in hepatocytes harvested at CT24 from control groups [showing induction levels of cells harvested at 12 hours for CXCL10 (middle) or 24 hours for CXCL11 (right) after induction with IFN-β]. (**B**) Circadian-dependent inflammatory response to IFN-β in Bmal1-silenced hepatocytes. Expression of inflammatory cytokines was measured by qPCR. Levels of cytokine mRNA were quantified (relative to GAPDH) and presented in relation to expression levels in hepatocytes harvested at CT24 from nontargeting or Bmal1 siRNA IFN-β–treated groups. (**C**) Inflammatory response to circadian-dependent LPS immune challenge. Schematic of induction with immune challenge (left). Expression of inflammatory cytokines was measured by qPCR. mRNA was isolated from synchronized hepatocyte cultures harvested at 4 hours after LPS treatment at either CT24 (light blue), CT36 (dark blue), or CT48 (gray). Levels of cytokine mRNA were quantified (relative to GAPDH) and are presented in relation to expression levels in hepatocytes harvested at CT24 from the control group. (**D**) Circadian-dependent inflammatory response to LPS in Bmal1-silenced hepatocytes. Expression of inflammatory cytokines was measured by qPCR. Levels of cytokine mRNA were quantified (relative to GAPDH) and presented in relation to expression levels in hepatocytes harvested at CT24 from nontargeting or Bmal1 siRNA LPS-treated groups. Data are mean ± SEM. *n* = 3 independent wells. Statistical significance was determined using a two-way ANOVA with post hoc pairwise comparisons. ****P < 0.0001, ***P < 0.001, **P < 0.01, and *P < 0.05.

It is well established that the response to an innate immune challenge is conditioned by the time of day at which cells are exposed to such a challenge. For example, previous work has shown that the response of human macrophages to lipopolysaccharide (LPS) varies according to the time of the day at which the cells are exposed to the antigen (*40*). To examine the induction of ISGs at different CTs, PHH cultures were synchronized; treated with IFN-β at CT24, CT36, or CT48; and harvested 12 and 24 hours after IFN-β addition (Fig. 4A). We found that transcript levels of *CXCL10* and *CXCL11* were induced to a greater extent when the IFN-β was added at CT36 compared to CT24 or CT48 (Fig. 4A and fig. S7C). Next, we silenced the expression of *Bmal1* in the human hepatocytes and showed an attenuation of the differences in circadian induction of *CXCL10* and *CXCL11* in response to IFN-β (Fig. 4B).

We next tested the time-dependent induction by LPS. PHH cultures were treated with LPS at CT24, CT36, or CT48, and expression levels of *IL6*, *IL1*β, and *TNF*α mRNA were assessed 2, 4, and 6 hours after exposure (Fig. 4C and fig. S7, D and E). The three pro-inflammatory cytokines were induced to a significantly greater extent when LPS was added at CT36 compared to exposure at CT24 or CT48 (Fig. 4B and fig. S4, D and E), these circadian induction differences were ablated when *Bmal1* was silenced in the hepatocytes (Fig. 4D and fig. S7, H and I).

We confirmed that these circadian differences were observed at the protein level by measuring the concentration of tumor necrosis factor–α (TNF-α), IL-6, and IL-1β secreted into the culture media (fig. S7F) and that exposure to LPS or IFN-β did not disturb the regular circadian profile (fig. S7G). *Bmal1* oscillations were observed with a reduction in the amplitude but rescued upon LPS or IFN-β removal (fig. S7G).

**Fig. 5.**
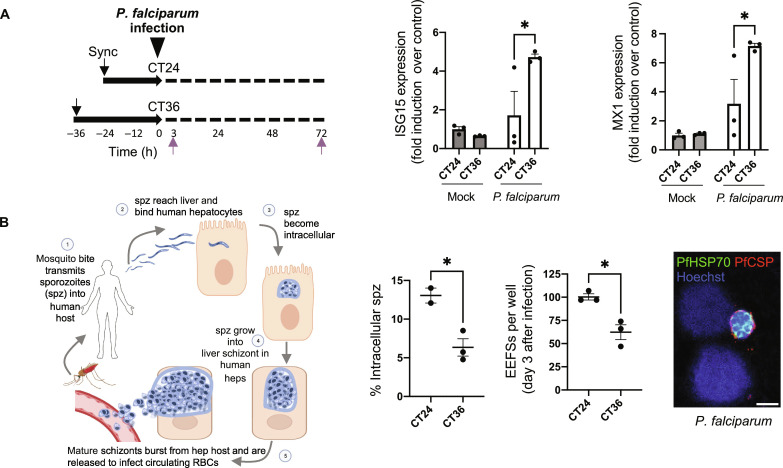
Circadian control of hepatocyte infection. (**A**) Infection of hepatocytes by malaria-causing *P. falciparum* sporozoites. Experimental workflow (left). mRNA was isolated from synchronized hepatocytes cultures harvested at 3 hours after infection at CT24 or CT36. Transcript levels of *ISG15* and MX1 mRNA were first normalized relative to *GAPDH* and are presented in relation to expression levels in mock-infected hepatocytes harvested at CT24 (right). Data are mean ± SEM. *n* = 3 independent wells. Statistical significance was determined using a two-way ANOVA. *P < 0.05. (**B**) Malaria life cycle schematic depicting the obligate initial liver stage expansion before egress to the blood (left). Quantification of intracellular parasites at 3 hours (corresponding to the transition from stage 2 to 3 in the left hand schematic) and 3 days (corresponding to schematic stages 3 and 4) after infection. Representative image of *P. falciparum* infection (parasite EEFs are identified by anti-HSP70 (green) and anti–circumsporozoite protein (red) staining) in human MPCCs at day 3 (right). Scale bar, 5 μm. Data are means ± SEM. *n* = 3 independent wells. Statistical significance was determined using an unpaired *t* test. *P < 0.05. (left). RBCs, red blood cells. PfCSP, *P. falciparum* circumsporozoite protein.

Last, we investigated circadian-dependent ISG induction by exposing PHH to a live hepatotropic human pathogen (Fig. 5B, right). PHH cultures were infected with malaria-causing *Plasmodium falciparum* parasites at CT24 or CT36 (Fig. 5A, left), and the ISG induction levels were examined 3 hours after exposure. Consistent with the IFN and LPS data, we found that *ISG15* and *MX1* were induced to a greater extent when the hepatocytes were infected at CT36 compared to CT24 (Fig. 5A, right). To further determine the impact on infection, we quantified the number of infected hepatocytes in the circadian-synchronized MPCC cultures. Strikingly, we found an inverse correlation between the ISG induction and the number of infections, with the percentage of infected hepatocytes being significantly higher at CT24 compared to CT36 (Fig. 5B). Reduced numbers of intracellular parasites at CT36 were consistently observed at 3 hours and 3 days after infection (Fig. 5B), pointing to an ISG-circadian–dependent mechanism controlling *Plasmodium* infection. Overall, our data demonstrated how circadian rhythms in human hepatocytes can influence the response to inflammatory stimuli and the outcome of infections.

## DISCUSSION

In this study, we describe the development of an experimental platform that enables the exploration of circadian-dependent genetic programs in PHHs. We observe that the hepatocyte autonomous clock acts independently of external signals and exhibits gene expression patterns that have been previously documented in human livers. By optimizing culture conditions, we demonstrated the dynamic state of human hepatocytes across a 48-hour period and how 24-hour cyclic fluctuations in their gene expression patterns can potentially influence liver physiology, drug metabolism, the inflammatory response, and the outcome of liver-stage *Plasmodium* infection.

Many molecular, physiological, and behavioral processes display distinct 24-hour rhythms that are directed by the central and peripheral circadian systems. Epidemiological evidence has documented a link between circadian regulation and human health, which has important consequences for disease risk and drug efficacy (*35*, *41*–*45*). On the basis of these observations, detailed descriptions of circadian rhythm–dependent processes displayed by different organs, and cells are necessary to gain insights into biological processes, develop diagnostic assays, and define disease risk prediction and new treatment protocols.

In the liver, this oscillatory genetic network regulates system-wide rhythmic gene expression programs, including components of fundamental metabolic pathways, energy expenditure, and other aspects of liver pathobiology (*46*). In vitro systems designed to define the impact of circadian rhythms on hepatic cell populations have been developed. These efforts include the use of hepatoma cells lines, which are known to exhibit defective patterns of metabolism (*47*, *48*) and immune responses (*49*, *50*), or primary mouse hepatocytes, which display a limited stable functional phenotype when cultured with standard in vitro systems (*30*, *51*). Previous studies have shown that although the core clock genes are quite conserved between species, among different tissue and species, there is considerable variation in the specific lists of clock-regulated genes and their corresponding physiological functions (*52*). Therefore, the incorporation of species- and tissue-specific cells, such as PHHs, is critical for the field.

To systematically study the impact of circadian rhythm on isolated hepatocyte gene expression, we performed cell-autonomous transcriptomics using PHHs. These experiments revealed the presence of 388 oscillating transcripts during the first 24 hours. The number of oscillating transcripts decreased during the next 24-hour period. However, the observation that the oscillations of the core clock genes also decrease over time in culture and that the *Bmal*-luc reporter expression exhibited a declining pattern that was restored after each new synchronization collectively indicate that the decrease in the number of genes detected may be a result of the gradual desynchronization of the cultures after 24 hours.

The circadian transcriptome revealed cycling genes related to drug metabolism, lipids, glucose, and cholesterol—all in line with previous observations made in patient samples or rodent models (*6*, *8*–*10*, *24*). In addition, we identified other oscillating pathways, such as (i) the retinoid pathway involved in the biosynthesis of all-trans retinoic acid, the major biologically active retinoid in vivo, and (ii) the pro-inflammatory signaling cascade activated by IFN. Previous research shows that cell-autonomous clocks within immune cells themselves direct variation in a large number of circadian parameters (*53*). However, fewer reports exist of circadian immune-related genes in nonimmune cells (*54*). By silencing the circadian clock master regulator *Bmal1* in these PHH cultures, we demonstrated the dysregulation of similar pathways identified in our circadian transcriptome.

The liver is a hub of energy metabolism and detoxification, the targeted host of different hepatotropic pathogens (including pathogens such as hepatitis B virus, hepatitis C virus, and malaria-causing parasites), and constantly encounters food-derived antigens and bacterial components translocated from the gut into the portal vein. These liver-specific aspects prompted us to focus on the circadian regulation of drug metabolism and the inflammatory response, and what we could observe of those patterns in our isolated human liver platform. It has been previously reported that ADME drug properties are controlled by circadian mechanisms, leading to altered bioavailability at different times of the day, and that the majority of Food and Drug Administration–approved drugs have targets that are regulated in a circadian pattern (*6*, *7*, *55*). Thus, integrating ADME properties (pharmacokinetics) and circadian knowledge regarding the activity of the targeted pathway (pharmacodynamics) could help to increase drug efficacy and diminish their side effects. In our PHH circadian transcriptome, we identified several ADME genes that oscillate in isolated, synchronized cultures including *CYP3A4*, a CYP450 that metabolizes almost 50% of all clinical drugs (*38*). Therefore, we infer that this circadian variation could have a substantial impact on the action of drugs with short half-lives and on the management of DDIs and should be taken into account during the design of safer protocols. DDI may occur when a patient is taking multiple medications simultaneously for one or more conditions. It is well described that the coadministration of CYP3A4-inducing or -inhibiting drugs requires close monitoring to avoid DDI and concomitant undesirable effects. Classic examples include the anti-HIV drug ritonavir (a CYP3A4 inhibitor) or the anti-tuberculosis drug rifampin (a CYP3A4 inductor). Here, we show how the induction level of CYP3A4 after dosing with rifampin depends on the dosing time. Therefore, this time-dependent response could be used as a strategy to modulate undesirable DDI by administrating rifampin or other CYP3A4-modulating drugs at the time where it causes the lowest induction of CYP3A4. Consistent with this hypothesis, after establishing CYP3A4’s circadian profile at the level of enzyme activity, we performed a proof-of-concept circadian-dependent toxicity analysis using two known hepatotoxicants, acetaminophen and atorvastatin. Both drugs can cause liver damage when administered at high doses (acetaminophen) or during chronic treatments (atorvastatin) due to the formation of toxic metabolites partially mediated by the activity of CYP3A4. Acetaminophen is a widely used analgesic and it is currently the most common cause of hepatic failure requiring liver transplantation. Atorvastatin is primarily used as a lipid-depleting agent in chronic treatments. In both cases, we demonstrated how toxicity correlated with the higher levels of CYP3A4 activity, thus confirming the CYP3A4-circadian hepatoxicity dependence. These experiments establish the feasibility to develop a treatment protocol to minimize the hepatotoxicity adverse effect of a subset of drugs.

Previous studies have demonstrated that the susceptibility of an animal to various infectious agents is time dependent and often correlates with differential induction of inflammatory cytokines (*17*, *56*, *57*). Our data support this concept by demonstrating differences in human hepatocyte sensitivity to LPS and IFN-β as well as to *Plasmodium* infection at different circadian phases. LPS is a highly antigenic molecule that is derived from Gram-negative bacteria and activates a plethora of inflammatory cascades via Toll-like receptor-4 . The temporal dependence of LPS-induced endotoxic shock and differential induction of inflammatory cytokines has been reported in mice. In vitro studies also observed a myeloid autonomous clock when challenged with LPS at different CTs after synchronization (*40*, *58*). In more recent work, Geiger and colleagues point to Bmal1 in hepatocytes as a key transducer in modulating susceptibility to the lethal effects of LPS (*59*). In our hepatocyte cultures, we also observed altered levels of inflammatory cytokine expression after induction with LPS at different times. An organism’s feeding process exposes the liver to food-derived bacterial components, such as LPS, that are translocated from the gut into the portal vein. Consequently, feeding rhythms will also produce rhythmic exposure to food-derived antigens and bacterial components. It has been previously shown that intestinal innate immunity exhibits circadian rhythms that anticipate pathogen exposure upon food intake (*54*, *60*) Therefore, hepatocytes may use a similar mechanism to anticipate the exposure to food-derived antigens and bacterial components during feeding. It is the reliable prediction of regular environmental changes that make circadian biology advantageous, such that these patterns could potentially serve as a mechanism to induce immune tolerance or a mechanism to achieve a more efficient immune response in the liver.

We also report differences in hepatocyte sensitivity to IFN at different circadian phases. IFN response is fundamental in the innate immune response to hepatotropic pathogens. IFN activates a signal transduction pathway leading to the induction of ISGs. Notably, we observed a down-regulation of several ISG in isolated PHHs after *Bmal1* expression was suppressed. This observation may explain why previous models observed that virus replication is enhanced in the absence of *Bmal1*, implying that low levels of *Bmal1* lead to increased herpes and influenza A viral infection (*36*) The fact that the induction of ISG in response to IFN was altered depending on the time of the stimulation could potentially lead to an innate response and infection outcomes that are variable across the circadian day, or after IFN treatment (*61*, *62*) In support of this theory, here we showed that the levels of ISG induction were significantly higher at CT36 versus CT24 and the number of Malaria-infected hepatocytes was the opposite (higher infection at CT24 versus CT36). In our system, at CT24 we observe the lowest sensitivity to IFN and LPS. However, we cannot rule out other factors, for example genes involved in lipid metabolism have been shown to be important for the malaria liver stage infection, and the circadian transcriptome obtained using our MPCC PHH revealed genes cycling related to lipid metabolism. Therefore, further investigations are needed to identify the malaria host-circadian factors.

These findings provide insights into the circadian control of inflammatory responses and hepatotropic pathogen infection in hepatocytes, which could have consequences for our understanding of the pathogenesis of inflammation and infectious diseases. Follow-up studies are necessary to understand the molecular mechanisms underlying the differential LPS- and IFN-mediated induction in hepatocytes and its implication in inflammatory processes and pathogen clearance, which could help in the development of anti-inflammatory and antimalarial and antiviral therapies.

Collectively, our data highlight the enabling capacity of in vitro systems to interrogate and probe circadian biology in a species- and tissue-specific cell (e.g., PHHs) and document how circadian oscillations in gene expression drive hepatocyte function in the context of drug metabolism, inflammation, and parasite infection.

## MATERIALS AND METHODS

### Methods details 

#### Cells

Cryopreserved PHH donors were purchased from BioIVT (BGW, UBV, GEB, WWL, JLO, LFQ, YWE, BDU, OQA, and FZQ) and Life Technologies (8373, 2096, 8350, 8300, 8391, and 3339). Both vendors permitted to sell products derived from human organs procured in the United States by federally designated Organ Procurement Organizations. Donor demographics are detailed in fig. S1E. Hepatocytes were maintained using standard hepatocyte medium [high-glucose Dulbecco’s modified Eagle’s medium (DMEM with l-glutamine, Corning)] with 10% (v/v) fetal bovine serum (FBS; Gemini), 1% (v/v) ITS+ (insulin/ human transferrin/selenous acid and linoleic acid) premix (BD Biosciences), glucagon (7 ng/ml; Sigma-Aldrich), dexamethasone (40 ng/ml; Sigma-Aldrich), 15 mM Hepes (Gibco), and 1% (v/v) penicillin-streptomycin (Corning). To synchronize the hepatocytes and visualize the cyclic expression of *Bmal1*, the cultures were placed in “circadian medium” [non-Hepes–buffered CO_2_-independent medium (COI; Thermo Fisher Scientific) with 10% (v/v) FBS (Gemini), 1% (v/v) ITS+ premix (BD Biosciences), and glucagon (7 ng/ml; Sigma-Aldrich) (fig. S1A)].

3T3-J2 male murine embryonic fibroblasts (gift of H. Green, Harvard Medical School) were cultured at <18 passages in fibroblast medium composing of DMEM with high glucose, 10% (v/v) bovine serum (Thermo Fisher Scientific), and 1% (v/v) penicillin-streptomycin (Corning).

#### Coculture and lentiviral treatment

PHHs were seeded on collagen-coated micropatterned plates as detailed previously (*27*). Hepatocytes were transduced with *Bmal1* luciferase expressing lentiviral pseudoparticles [pLenti6-B4B2-Bmal1-dLuc; (*26*); provided by M. Young] 4 hours after seeding in the presence of polybrene (4 μg/ml) by spinoculation for 45 min at 1000*g*. Twelve to 16 hours after transduction, hepatocytes were washed and 3T3-J2 fibroblasts were subsequently added to the cultures. One week after transduction, *Bmal1* luciferase expression reached a measurable level and remained stable over time.

#### Coculture and siRNA treatment

PHHs were seeded on collagen-coated micropatterned plates as detailed previously (*27*). siRNA oligonucleotides were added to patterned hepatocytes after washing off the unbound hepatocytes. ON-TARGETplus SMARTpool siRNA oligonucleotides (Dharmacon) were delivered to hepatocytes by using RNAiMAX Transfection Reagent (Thermo Fisher Scientific) per the manufacturer’s protocols at a final concentration of 100 nM in antibiotic-free DMEM media supplemented with 10% FBS (final volume of 100 ml). Duplicate or triplicate wells containing hepatocytes were exposed to siRNA duplexes overnight for 20 to 24 hours and subsequently surrounded with 3T3-J2 fibroblasts and cultured in supplemented hepatocyte media.

#### RNA-seq processing and analysis

Total RNA was extracted using TRIzol (Thermo Fisher Scientific) and purified using the RNeasy Mini Kit (QIAGEN) according to the manufacturer’s instructions. Samples were deoxyribonuclease–treated. RNA integrity was determined using an Agilent Fragment Analyzer, and purity and quantity were determined using a Thermo Scientific NanoDrop 1000 Spectrophotometer. High-throughput RNA-seq libraries were prepared as previously described (*63*) using 100 ng of total RNA input and sequenced on a NovaSeq6000 using 50-nt paired-end sequencing.

In the time series experiment, gene expression was quantified using RSEM version 1.3.1 and STAR version 2.7.1a alignment to a transcriptome derived from the human hg38 primary assembly and ensembl version 98 annotation (*64*). Transcripts per million expression data with a +1 offset was transformed to log_2_ space and assembled using Tibco Spotfire Analyst 7.6.1, resulting in a data matrix with two or three replicates for 17 time points at 3-hour intervals. The average and variance across all replicates were calculated, and genes with average expression less than 0.2 and variance less than 0.05 were excluded from analysis. These data were used as input to JTK_CYCLE (*65*), and the software was executed according to the author’s instructions. Separate runs were performed for 24- to 72-hour and the 24- to 48-hour time spans, and oscillating genes were defined as those having BHQ values less than 0.2, 0.1, or 0.05. Heatmaps and scatter plots of oscillating genes were prepared in Tibco Spotfire Analyst 7.6.1. Heatmaps used row-centered data calculated from averaged replicates using the STANDARDIZE function of Microsoft Excel and *x*-axis ordering using the JTK_CYCLE Lag statistic. Functional annotation of oscillating gene lists was done using the overlap utility of MSigDB (*34*).

For the *Bmal1* knockdown experiment, gene expression was quantified using Salmon version 1.3.0 (*66*) with a transcriptome target consisting of human and mouse transcripts. Human transcripts were derived from the hg38 primary assembly and the ensembl version 101 annotation, and mouse transcripts were derived from the mm10 primary assembly and the ensembl 101 annotation (*64*). Gene level summaries were prepared using tximport version 1.18.0 (*67*) running under R version 4.0.3 (R Core Team 2021; www.R-project.org.). Differential expression analysis was done for human genes with DESeq2 version 1.32.0 (*68*, *69*), and differentially expressed genes were defined as those having an absolute apeglm (*70*) log_2_ fold change greater than 1 and an adjusted *P* value less than 0.05. Data parsing and clustering was done using Tibco Spotfire Analyst 7.6.1. Preranked GSEA (*71*) was done using javaGSEA version 4.1.0 using the Wald statistic for human protein-coding genes as ranking metric and MSigDb version 7.2 (*34*, *72*) gene sets.

#### RNA isolation and RT-PCR

Upon media removal, MPCCs were lysed and homogenized in TRIzol (Thermo Fisher Scientific). RNA was isolated via chloroform extraction and further purified with the RNeasy MinElute Cleanup Kit (QIAGEN). cDNA was synthesized utilizing SuperScript II (Thermo Fisher Scientific), and qPCR was performed using PowerUp SYBR Green Master Mix (Thermo Fisher Scientific) in a Bio-Rad CFX96 Real-Time System according to the manufacturer’s instructions (*37*). The primers sequences used to detect mRNA levels are listed in table S2. Relative mRNA quantification was calculated with the DDCt (Delta-Delta Ct) method, using *gapdh* as housekeeping gene.

#### Bmal Real-time monitoring

Luminescence of transduced hepatocytes with *Bmal1*-luc reporter was monitored in real time at 20- to 25-s sampling resolution (integration time) by light emission of luciferase over 2 to 3 days in “circadian medium” (COI + l-glutamine + FBS + PS + ITS + glucagon) supplemented with d-luciferin substrate (100 to 150 μg/ml; Biosynth). Luminescence was determined using a Synergy Neo2 HTS Multi-Mode Microplate Reader, BioTek. Source of plasmid is noted above.

#### Biochemical assays

The activity of CYP3A4 in MPCCs was monitored using the cell-based P450-Glo CYP3A4 Assay with luciferin–isopropyl acetal (IPA) (Promega) according to the manufacturer’s instructions (*37*).

Albumin levels were measured by enzyme-linked immunosorbent assay (ELISA). Cultures supernatants were collected and stored at 20°C. Diluted supernatants were incubated with immobilized anti-human albumin antibodies (Bethyl, catalog no. A80-129, RRID: AB_67016) for 2 hours at room temperature or overnight at 4°C. After washing, plates were incubated with horseradish peroxidase–conjugated anti-human albumin antibodies (Bethyl, catalog no. A80-129P, RRID: AB_67023) for 1 hour at room temperature and developed with tetramethylbenzidine (Thermo Fisher Scientific) as per the manufacturer’s protocols. Albumin content was determined on the basis of the standard curves included on each plate (*37*).

#### Drug, LPS, and IFN treatment of MPCCs

MPCCs at different CTs (CT24, CT36, and CT48) were dosed with rifampin (Sigma-Aldrich) at 2.5 μM or atorvastatin (Cayman) ranging from 10 to 600 μM or acetaminophen ranging from 0.5 to 50 mM or LPS (InvivoGen) ranging from 10 to 100 ng/ml or IFN-β (R&D Systems) ranging from 100 to 1000 U/ml.

#### Plasmodium infection of MPCC

*P. falciparum* NF54 or NF175 sporozoites used for infections were isolated via hand dissection of salivary glands of *Anopheles stephensi* female mosquitoes bred and infected at Institut Pasteur (Paris, France) or Radboud University Medical Center (Nijmegen, Netherlands). For transcript analysis, 96-well format MPCCs synchronized at different CTs (CT24 and CT36) were exposed to 20,000 sporozoites per well or equivalent material from noninfected mosquito salivary glands. Pools of three wells were harvested 3 hours later and processed for RT-PCR.

For quantification of intracellular parasites, synchronized MPCCs were infected with 20,000 or 100,000 sporozoites and fixed in 4% paraformaldehyde at 3 hours or 3 days after infection, respectively. Parasites were detected by immunofluorescence assay using *P. falciparum*–specific antibodies [*P. falciparum* circumsporozoite protein (PfCSP; 1:200 or PfHSP70, 1:200] as described (*27*, *73*). The number of parasites per well was scored using ImageJ software. Images were captured on a PerkinElmer Opera Phenix system or Zen-ApoTome inverted wide-field microscope using 20× objectives.

#### RealTime-Glo MT Viability Assay

Cell viability was measured using the RealTime Glo MT Cell Viability Assay (catalog no. G9711, Promega, Madison, WI) in situ. MT Cell Viability Substrate and NanoLuc Enzyme were diluted 1:100 in media, and 10 μl was added to each well for a final dilution of 1:1000. Viability was measured using a luminescent read with 1-s integration time.

#### Enzyme-linked immunosorbent assay

An ELISA method was used for measurement of serum IL-1β, IL-6, and TNF-α using commercially available ELISA kits (catalog nos. DLB50, D6050, and DTA00D, R&D Systems, Minneapolis, MN, USA).

### Quantification and statistical analysis

Polar histograms were generated using MATLAB. Each bin (2 hours) corresponds to 0.524 radians or 30°. Longitudinal measurements (phase, amplitude, and period) were generated in R using the JTK package.

Statistics were determined with a one-way or two-way analysis of variance (ANOVA) test with multiple comparisons using GraphPad Prism software. Statistical significance was considered for *P* values below 0.05 (*P < 0.05, **P < 0.01, ***P < 0.001, and ****P < 0.0001). Values in bar graphs represent means ± SEM, and the number of independently performed experiments (*n*) is indicated in the corresponding figure legend.
