# Supplementary material for: Autonomous circadian rhythms in the human hepatocyte regulate hepatic drug metabolism and inflammatory responses
Source: Sci Adv. 2024 Apr 24;10(17):eadm9281. doi: 10.1126/sciadv.adm9281 (PMC11042741; doi:10.1126/sciadv.adm9281)
Supplement: Supplementary file 1 — Figs. S1 to S7 Tables S1 and S2 [file sciadv.adm9281_sm.pdf]

Science Advances
AAAS

Supplementary Materials for

Autonomous circadian rhythms in the human hepatocyte regulate hepatic drug metabolism and inflammatory responses

Sandra March *et al.*

Corresponding author: Sangeeta N. Bhatia, sbhatia@mit.edu; Sandra March, smarch@mit.edu

Sci. Adv. **10**, eadm9281 (2024)
DOI: 10.1126/sciadv.adm9281

This PDF file includes:

Figs. S1 to S7
Tables S1 and S2

A

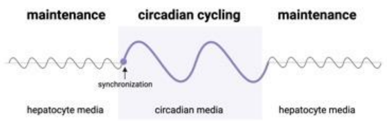

Hepatocyte media: DMEM (containing L-glutamine) + FBS (10%) + PS + ITS + Glucagon + Dexamethasone

Circadian media: Non-HEPES-buffered CO2 Independent Medium (COI) + L-glutamine + FBS (10%) + PS + ITS + Glucagon

B

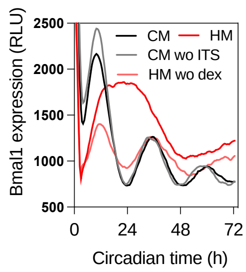

C

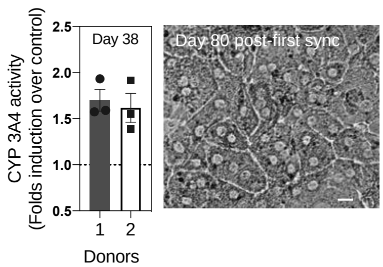

D

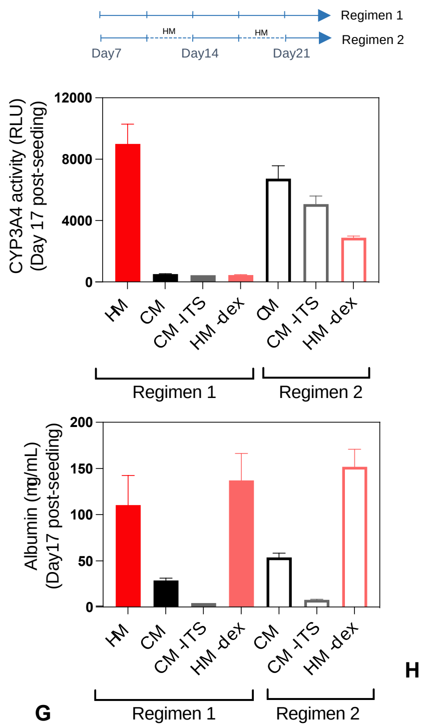

E

Lot	Sex	Race	Age	Period (h)
GEB	M	AA	48	23.3
BGW	M	CAU	50	23.8
UBV	M	CAU	57	24
8373	F	CAU	26	24.3
2096	M	CAU	47	25.8
WWL	M	CAU	13	26.2
8350	M	CAU	26	26.7
8300	M	CAU	31	26.7
JLO	F	CAU	58	26.8
LFQ	M	CAU	51	27.2
8391	M	CAU	62	27.5
YWE	M	CAU	29	27.5
BDU	F	CAU	50	27.6
OQA	M	AI	62	27.8
8339	F	AA	31	28
FZQ	F	CAU	69	28

M= Male, F= Female, AA= African American, CAU=Caucasian, AI = American Indian

F

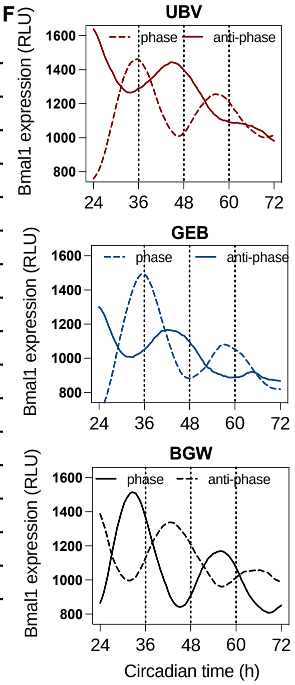

G

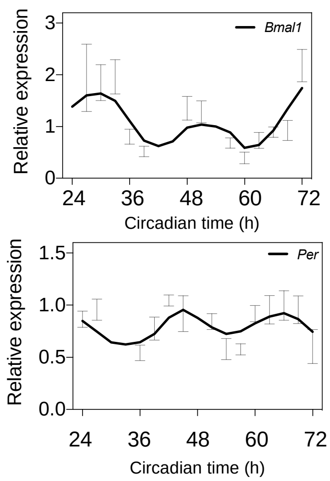

H

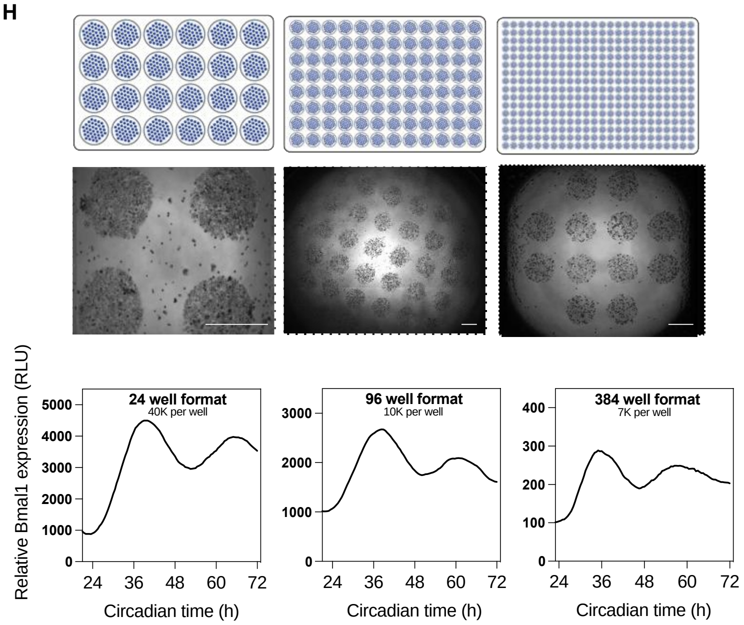

Figure 1 Supplemental:

Assessment of different media conditions and regimens.
(A) Schematic representation and composition of hepatocyte media utilized for hepatocyte maintenance, and circadian media utilized for synchronization and visualization of circadian oscillations.

(B) Real-time cycling of PHH cultures was monitored to compare various media conditions and their effect on *Bmal1* cycling; circadian media (black), circadian media minus ITS (gray), hepatocyte media (red), hepatocyte media minus dexamethasone (DEX) (light red).

(C) CYP3A4 fold induction activity in PHH (left). Representative primary human hepatocytes 80 days post-first synchronization (right). Scale bar = 10 μm.

(D) CYP450 activity and albumin secretion of PHH at day 17 post-seeding using different media compositions of hepatocyte media and regimens. Regimen 1: continues the use of the same media. Regimen 2: combination of standard hepatocyte media (HM) for 3.5 days followed by a medium exchange using a different medium (Circadian media "CM" or Circadian media without ITS "CM -ITS" or Hepatocyte media without Dexamethasone "HM-dex").

(E) *Donor demographics*
Table of 16 primary human hepatocyte donors including demographics (sex, race, and age) and period measurements using JTK software.

(F) *Anti-phasic Bmal1 expression of PHH cultures from 3 donors*
Synchronization of two sets of PHH cultures from 3 different donors [UBV (red), GEB (blue), and BGW (black)] was performed with a circadian medium change 12 hours apart, allowing them to free-run under constant conditions for 72 hours.

(G) *qPCR of Bmal1 and Per2 genes.*
Samples were taken every 3 hours for 48 hours post-synchronization and relative expression (normalized to housekeeper, *Gapdh)* was measured.

(H) *Validation of oscillating PHH cultures in different formats.*
Circadian rhythm was able to be monitored in 24-well (left), 96-well (middle), and 384-well (right) formats. Scale bar = 500 μm.

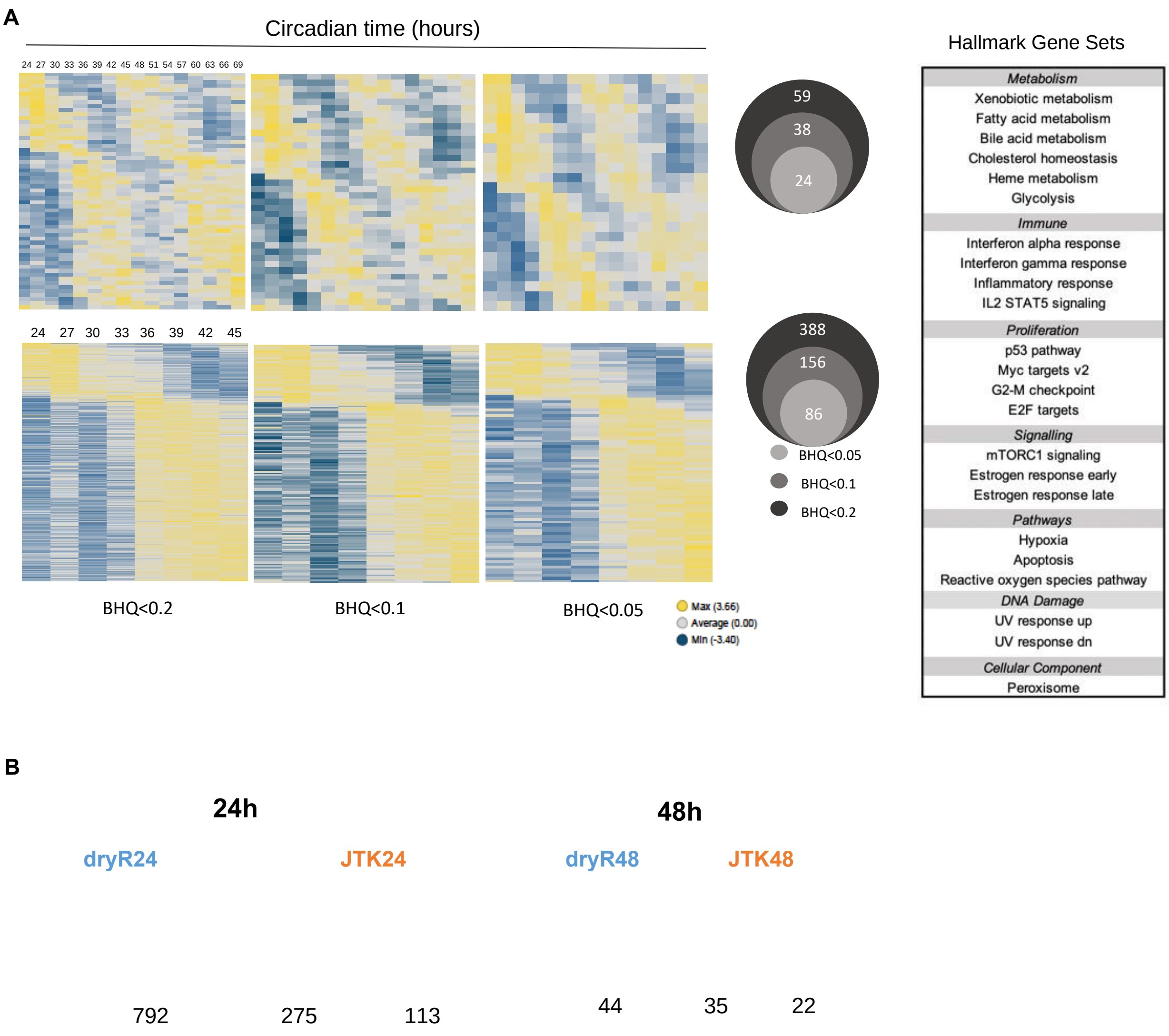

Figure 2 Supplemental:

(A) *Heat map representation of oscillating transcripts.*
Heat map representation of oscillating transcripts ordered by the time of the oscillation (columns), during a period of 48 hours (**top, left**) and 24 hours (**bottom, left**). Heat maps are ordered by threshold level – BHQ<0.2 , BHQ<0.1, and BHQ<0.05. Each vertical column represents a time point (3h resolution). Each row is a cycling transcript, colored based on the expression intensities, low (blue) and high (yellow). Expression values are mean-normalized for each gene and are ordered by the peak of expression. The number of transcripts is organized in concentric circles based on BHQ thresholds for every 48h (**top, middle**) and 24h (**bottom, middle**).

Gene Set Enrichment Analysis (GSEA) using a hallmark gene set collection as part of MSigDB (Molecular Signatures Database). Significant above 1.3 FDR-q = p> 0.05. All sets included in the table have an FDR-q above 1.3 (Input data 24h, BHQ <0.2) (**right**).

(B) *Algorithms comparison.*
Venn diagrams representing overlaps among the rhythmic transcripts identified by dryR or JTK algorithm at the 24h **(A)** or 48h **(B)** time point.

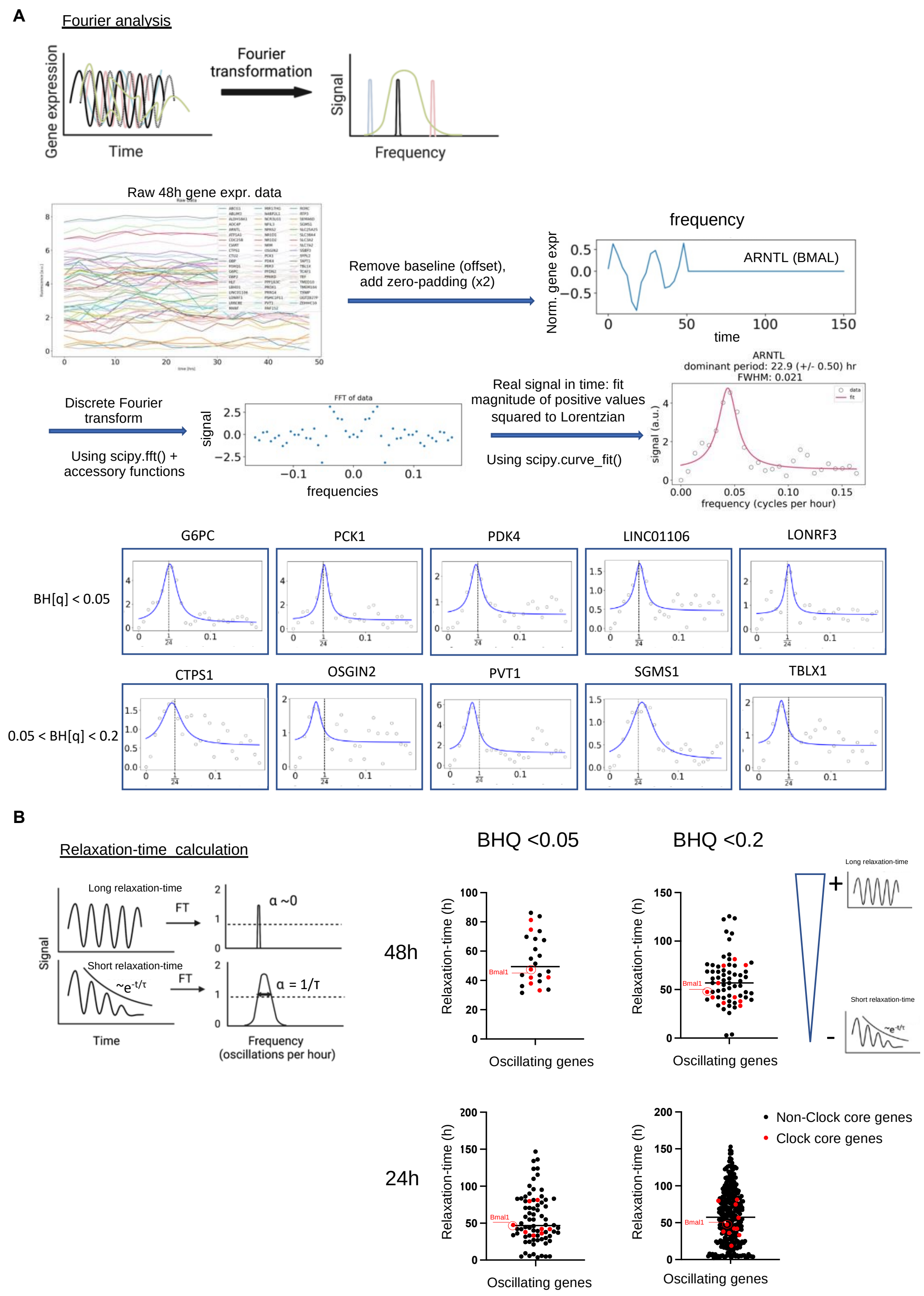

Figure 3 Supplemental:

(A) *Quantitative demonstration of circadian rhythmic activity via Fourier analysis*. Fourier analysis of ARNTL (*Bmal1*) expression in synchronized cultures, demonstrating a dominant period of approximately 24h (**top panel**). Fourier analysis of representative genes identified in our

circadian transcriptome, showing association between BH[q] score and noise level in Fourier domain (**bottom panel**).

(B) *Relaxation-time calculation.* Schematic of idealized signals and their Fourier analysis. A perfect circadian signal would result in a long relaxation-time value, while a decaying, imperfect circadian signal would result in a shorter relaxation-time value.

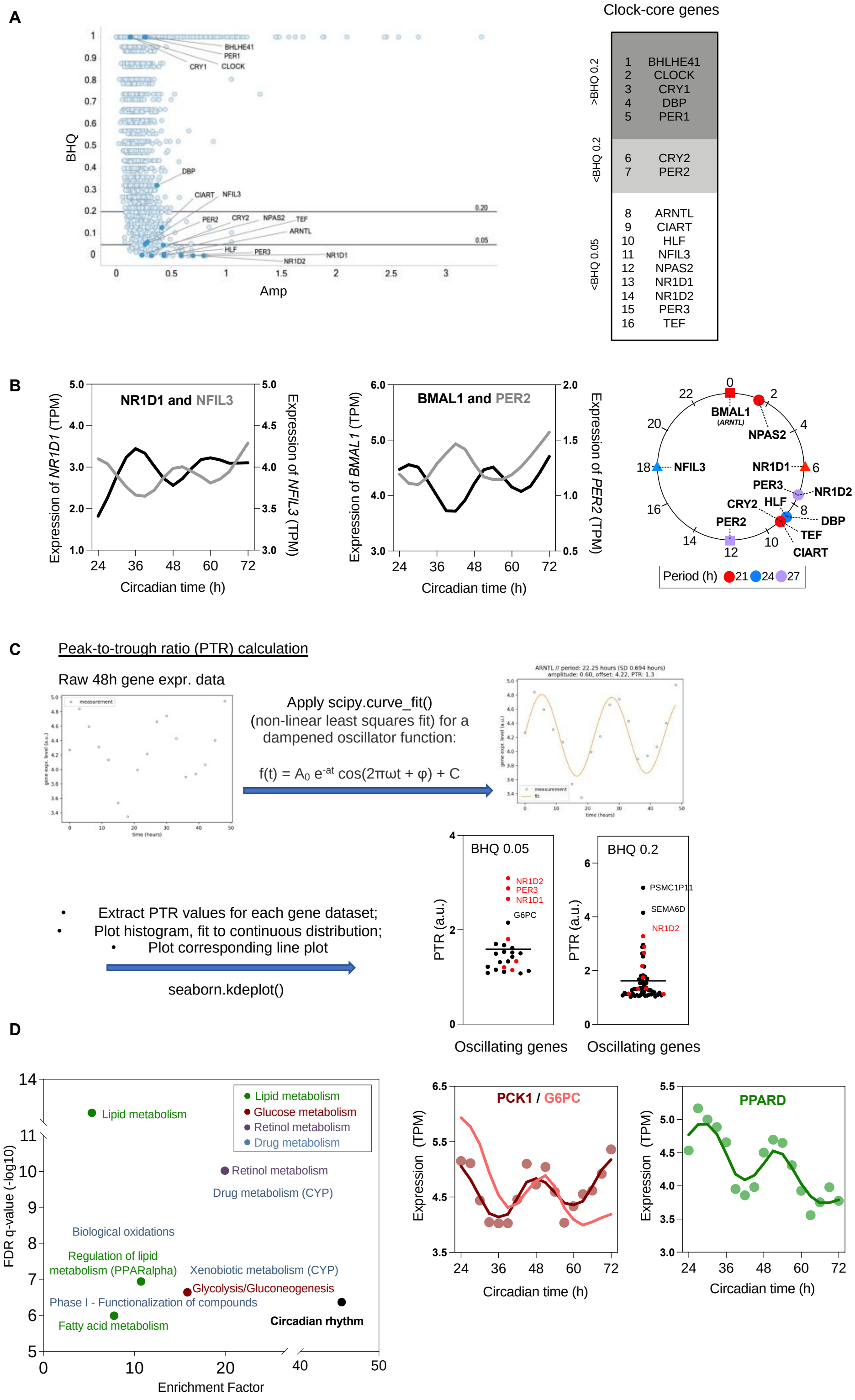

Figure 4 Supplemental:

(A) Threshold plots of oscillating genes (BHQ and amp). Dark blue (core clock genes). X-axis = amplitude, Y-axis = BHQ thresholds (**left**). Core clock genes are categorized by BHQ expression levels. 5 core clock genes that fall under BHQ>0.2 are not included for analysis (**right**).

(B) Left, middle *Representative core clock genes as cycling transcripts from bulk transcriptomic data. Bmal1* (dark gray, square) and *Per2* (light gray, square) show anti-phasic expression. *Nr1d1* (dark gray, triangle) and *Nfil3* (light gray, triangle) show anti-phasic expression. Expression shown in transcripts per million (TPM).
Right. *Distribution of the peak phases of core clock components.*
Peak phase of *Bmal1* (*Arntl*) was set as 0 and peaks of other genes were normalized to *Bmal1* expression peak and period. Core clock genes are colored by their expression periods – 21 hours (red), 24 hours (blue) and 27 hours (purple).

(C) *Peak to trough ratio (PTR) for rhythmic genes.* Input data 48h, BHQ <0.05 (**left**) and BHQ <0.2 (**right**). Red dots indicate core clock genes.

(D) ***Left.*** *GSEA plot of canonical gene set collections.*
Gene Set Enrichment Analysis using a canonical gen sets collection (KEGG and Reactome) as part of MSigDB (Molecular Signatures Database). (Input data 24h, BHQ <0.2). Significant above 1.3 FDR-q = p< 0.05. Identification of 4 groups of liver-specific functions related to glucose (red), lipid (green), retinol (purple) and drug metabolism (blue).
Right. *Representative oscillating genes*
Expression of *Pck1* (maroon), *G6pc* (light red) and *Ppard* (green) in transcripts per million (TPM).

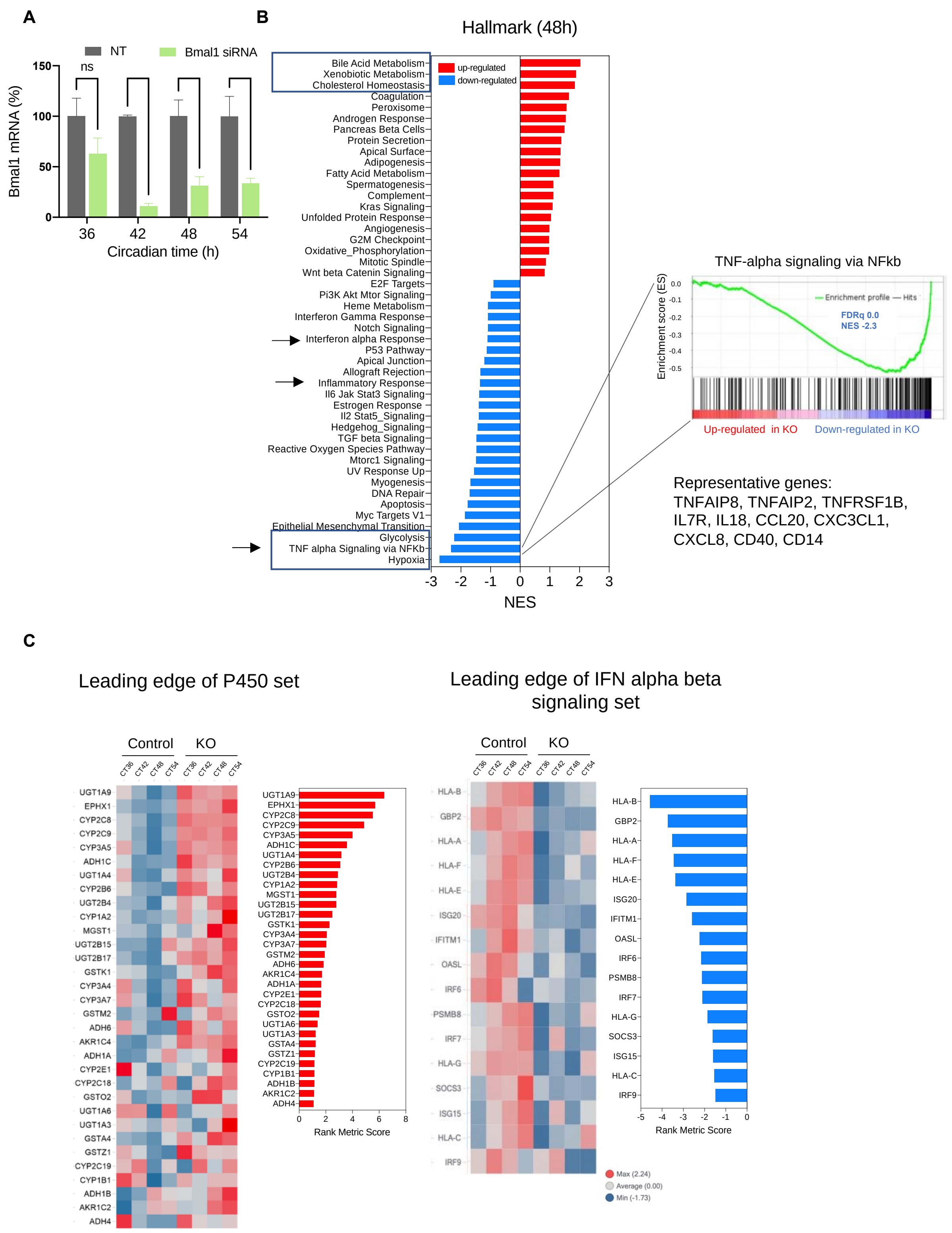

Figure 5 Supplemental:

(A) *Bmal1 silencing efficiency.*

Bmal1 silencing efficiency was measured by qRT-PCR at 6-hour intervals at CT12, CT18, CT24, and CT30. Si-RNA targeted samples (green) were compared to irrelevant non-targeted controls (NT) (gray) at each timepoint. $^{**}p < 0.01$. $^{*}p < 0.05$.

(B) Gene Set Enrichment Analysis using a hallmark gene sets collection as part of MSigDB (Molecular Signatures Database). Showing all statistically differentially expressed gene sets that were up (red)- or- down (blue) regulated in *Bmal1* knock-down cultures (**left**). TNF-α signaling via NFκB enrichment profile including a list of representative genes enriched in this set (**right**).

(C) Leading edge list of P450 set (**left**) and IFN alpha beta signaling (**right**) sets.

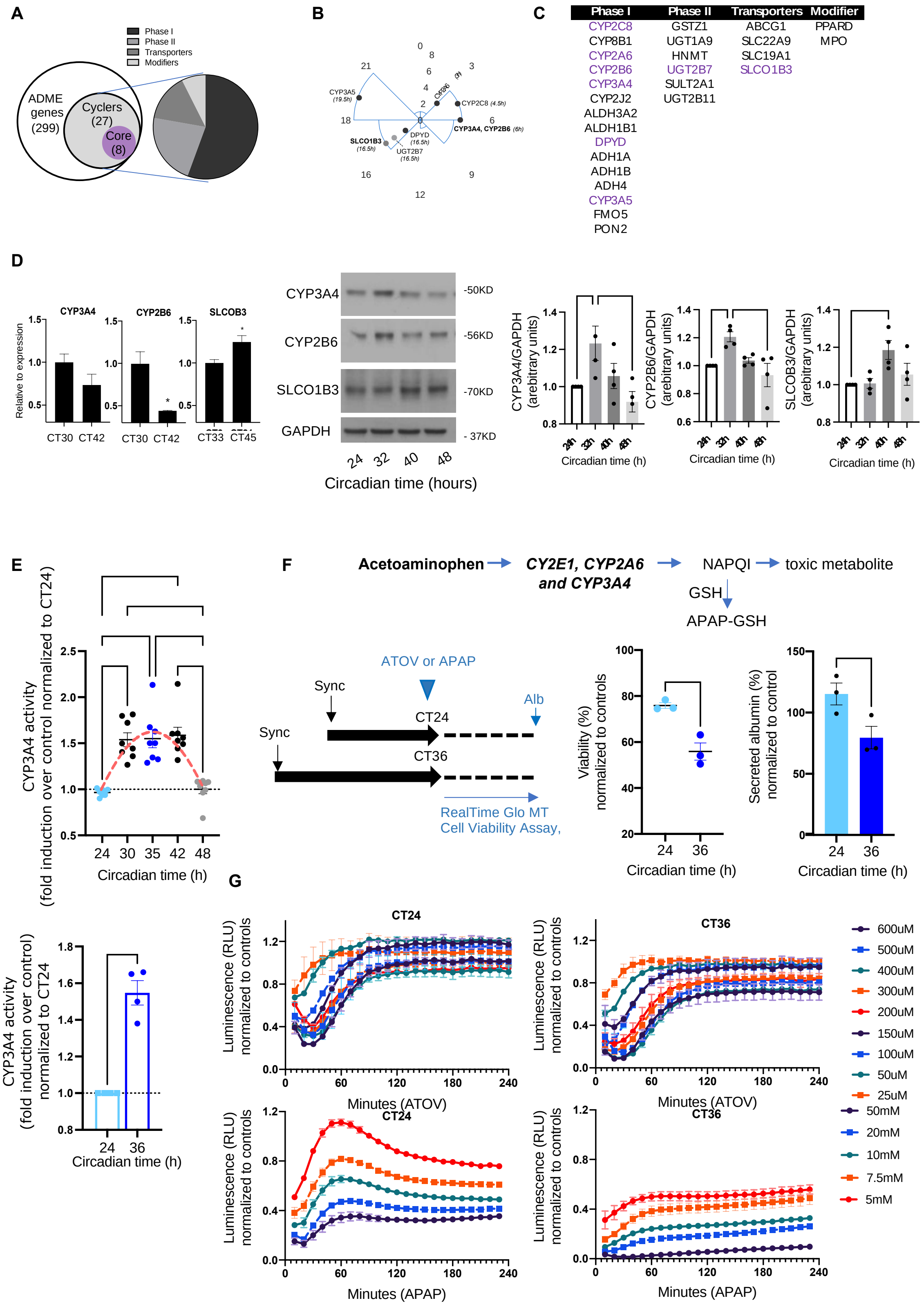

Figure 6 Supplemental:

(A) *Stages of drug uptake involving identified oscillating transcripts.*

Identification of 27 cycling ADME genes, out of 299 total transcripts, and classification of the oscillating ADME genes into four categories: Phase I and II metabolism enzymes, transporters, and modifiers. Of these genes, 8 belonged to the Core ADME genes (purple).

(B) *Polar histogram of ADME oscillating gene expression peaks.*
Polar histogram represents the frequency distribution of the peaks of the identified oscillating ADME genes. Cycling core drug metabolism enzymes are highlighted on the histogram, color corresponding to their respective ADME phases from figure 3B. Peak phase of Bmal1 was set at t=0, for comparison.

(C) List of the oscillating ADME genes classified into four categories: Phase I and II metabolism enzymes, transporters, and modifiers. 8 belonged to the Core ADME genes (purple).

(D) QPCR validation of *CYP3A4*, *CYP2B6*, and *SLCO1B3* (**left**). Western blot of protein extracts taken every 8 hours with antibodies for representative drug metabolism enzymes cytochrome P450 CYP3A4 and CYP2B6, and transporter SLCO1B3, along with housekeeper GAPDH (**middle**). Western blot quantifications of 3-4 independent experiments (**right**).

(E) *Impact of rifampin induction on CYP3A4 activity.*
Synchronized cultures at CT24, CT30, CT35, CT42 and CT48 (**top**) were dosed with rifampin (2.5μM) and 24h later CYP3A4 activity was measured via luminogenic assay. Statistical significance was determined using a two-way ANOVA with post hoc pairwise comparisons. ****p < 0.0001, **p < 0.01. Synchronized cultures at CT24 and CT36 **(bottom)** were dosed with rifampin (2.5μM) and 24h later CYP3A4 activity was measured via luminogenic assay. Data are mean ± s.e.m., $n = 4$ independent experiments. **$P < 0.01$.

(F) Experimental workflow (**left**). Schematic showing the metabolism of acetaminophen by CYP3A4 and its by-products (**top right**). ATP-based real-time viability assay to determine CYP3A4-metabolized toxicity of acetaminophen in PHH cultures synchronized 12h apart (CT24 vs CT36). Acetaminophen at 5mM was added and real-time viability was measured for 4 hours. Albumin levels 6 hours after dosing with Acetaminophen (bottom right). *p < 0.05.

(G) ATP-based real-time viability assay to determine CYP3A4-metabolised toxicity of scaling doses of atorvastatin (**top panel**) and acetaminophen (**bottom panel**) in PHH cultures synchronized 12h apart (CT24 vs CT36). Drugs were added to media and real-time viability was measured for 4 hours. Luminescence counts were divided by the respective DMSO controls and plotted versus time.

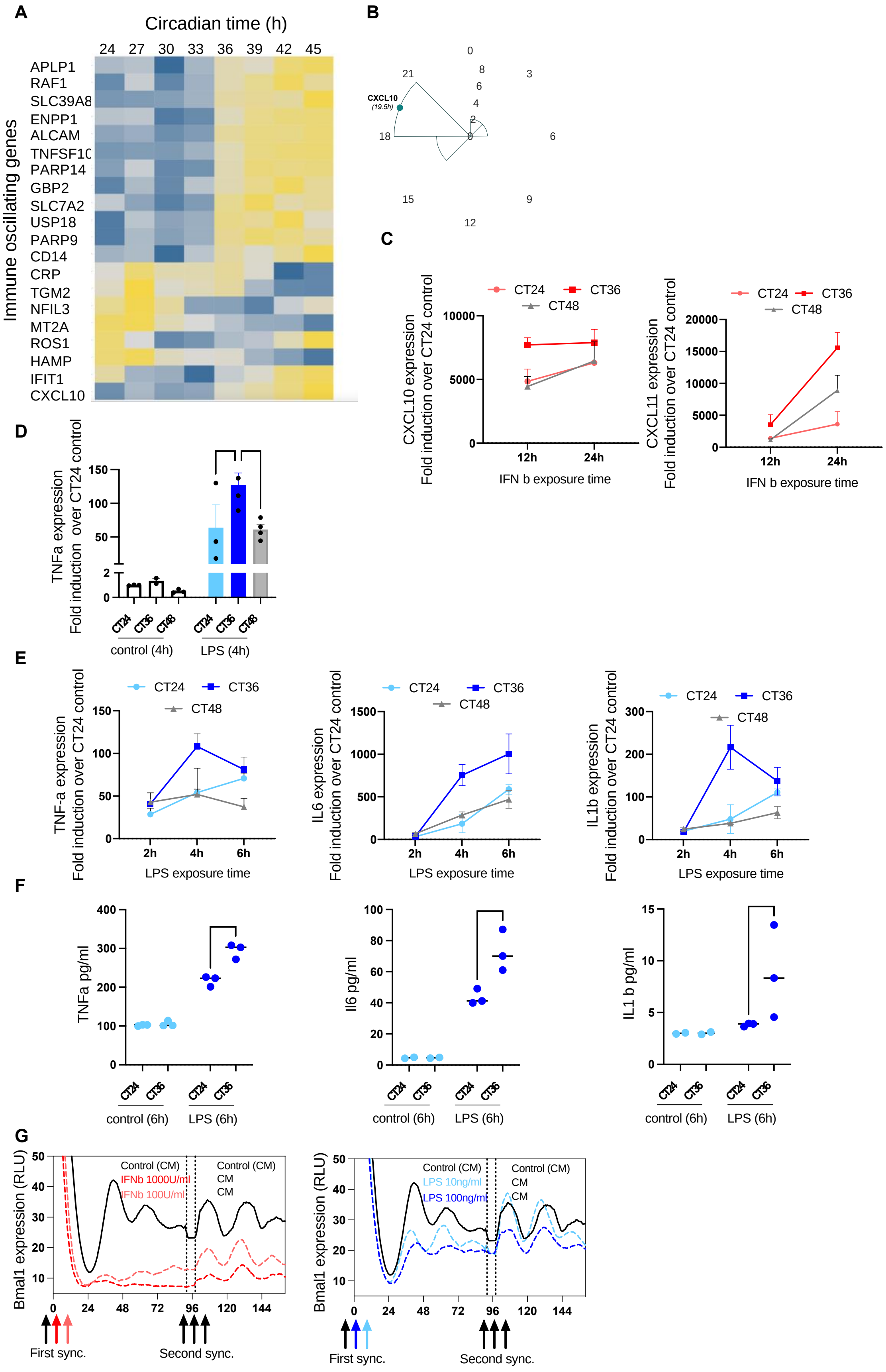
A
Circadian time (h)
24 27 30 33 36 39 42 45
Immune oscillating genes
APLP1
RAF1
SLC39A8
ENPP1
ALCAM
TNFSF10
PARP14
GBP2
SLC7A2
USP18
PARP9
CD14
CRP
TGM2
NFIL3
MT2A
ROS1
HAMP
IFIT1
CXCL10
B
CXCL10
(19.5h)
C
CT24 CT36 CT48
CXCL10 expression
Fold induction over CT24 control
12h 24h
IFN b exposure time
CXCL11 expression
Fold induction over CT24 control
D
TNFa expression
Fold induction over CT24 control
CT24 CT36 CT48
control (4h)
LPS (4h)
E
TNF-a expression
Fold induction over CT24 control
IL6 expression
Fold induction over CT24 control
IL1b expression
Fold induction over CT24 control
2h 4h 6h
LPS exposure time
F
TNFa pg/ml
Il6 pg/ml
IL1 b pg/ml
control (6h)
LPS (6h)
G
Bmal1 expression (RLU)
Control (CM)
IFNb 1000U/ml
IFNb 100U/ml
CM
LPS 10ng/ml
LPS 100ng/ml
First sync.
Second sync.

H

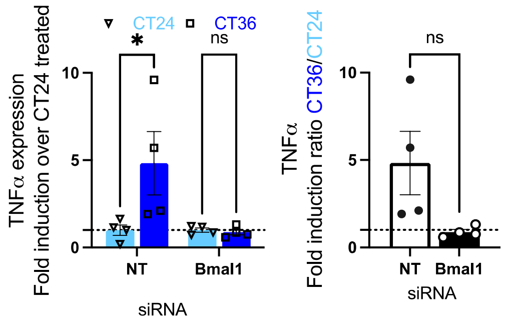

I

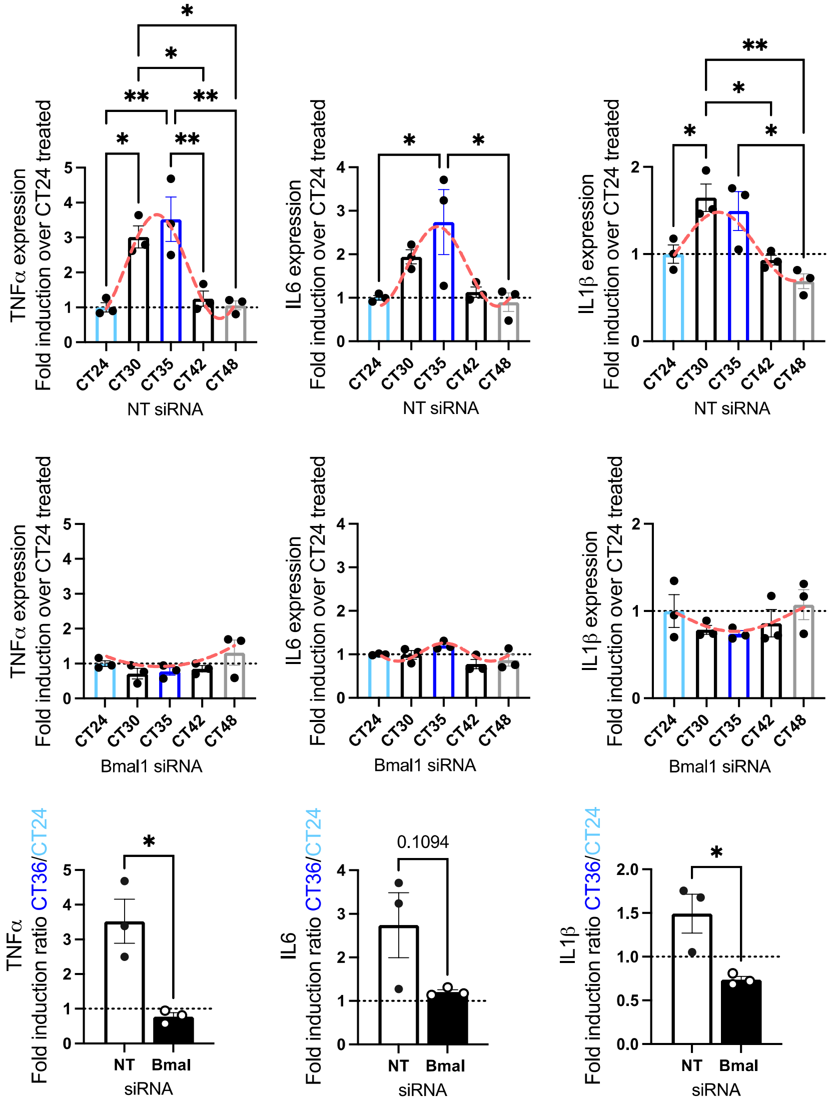

Figure 7 Supplemental:

(A) *Transcriptomic analysis of immune oscillating transcripts.*

Phase-sorted heat map of immune oscillating genes over a 24-hour period. Each vertical column represents a time point (3h resolution). Each row is a cycling transcript, colored based on the expression intensities, low (blue) and high (yellow) (BHQ <0.2). Expression values are mean-normalized for each gene and are ordered by peak of expression.

(B) *Polar histogram of immune oscillating gene expression peaks.*
Polar histogram representing the frequency distribution of the expression peaks of identified oscillating immune genes. Each section (45°) corresponds to 3 hours and each concentric circle corresponds to 2 oscillating genes. Peak expression of *Cxcl10* is highlighted on the polar histogram, at 19.5h.

(C) mRNA was isolated from synchronized hepatocytes cultures harvested at 12h and 24h after IFN-β treatment at either CT24 (light-red), CT36 (dark-red), or CT48 (gray). Levels of cytokine mRNA were quantified (relative to GAPDH) and presented in relation to expression levels in hepatocytes harvested at CT24 from control groups. Data are mean ± s.e.m., n = 3 independent wells.

(D) Expression of inflammatory cytokines was measured by q-PCR. mRNA was isolated from synchronized hepatocytes cultures harvested at 4h after LPS treatment at either CT24 (light-blue), CT36 (dark-blue), or CT48 (gray). Levels of *TNF-alpha* mRNA were quantified (relative to *GAPD*H) and are presented in relation to expression levels in hepatocytes harvested at CT24 from the control group.
Data are mean ± s.e.m., n = 3 independent wells. Statistical significance was determined using a two-way ANOVA with post hoc pairwise comparisons. ****$p < 0.0001$, **$p < 0.01$. *$p < 0.05$.

(E) mRNA was isolated from synchronized hepatocytes cultures harvested at 2h, 4h, or 6h after LPS treatment at either CT0 (light-blue), CT12 (dark-blue), or CT24 (gray). Levels of *TNF-α, IL6*, and *IL1-β* mRNA were quantified (relative to *GAPDH*) and are presented in relation to expression levels in hepatocytes harvested at CT0 from the control group (showing kinetics of induction of cultures harvested at 2h, 4h, and 6h post-LPS induction). Data are mean ± s.e.m., n = 3 independent wells.
(F) Supernatant protein level of *TNF-α, Il-6* and *Il-1β* of synchronized hepatocytes cultures after 6h of LPS stimulation. Data are mean ± s.e.m., n = 3 independent wells. Statistical significance was determined using a two-way ANOVA with post hoc pairwise comparisons. ****$p < 0.0001$, **$p < 0.01$. *$p < 0.05$.

(G) *Influence of immune challenges on Bmal1 expression.*
At the time of synchronization, hepatocytes transduced with *Bmal1-luc* reporter, were dosed with IFN-β (**left**) and LPS (**right**). *Bmal1* real-time expression was monitored over a period of 96h. A second synchronization was performed with a media exchange to all 3 groups. *Bmal1* real-time expression was monitored for an additional 72h.

(H) *Circadian-dependent inflammatory response to LPS in Bmal1-silenced hepatocytes.*
Expression of inflammatory cytokines was measured by q-PCR at CT24 and CT36. Levels of *TNFα* mRNA was quantified (relative to *GAPDH*) and presented in relation to expression levels

in hepatocytes harvested at CT24 from NT (non-targeting) or *Bmal1* siRNA LPS-treated groups (**left**). Fold induction ratio (**right**).

(I) *Circadian-dependent inflammatory response to LPS in Bmal1-silenced hepatocytes.* Expression of inflammatory cytokines was measured by q-PCR at CT24, CT30, CT35, CT42 and CT48. Levels of cytokines mRNA were quantified (relative to GAPDH) and presented in relation to expression levels in hepatocytes harvested at CT24 from NT (non-targeting) or Bmal1 siRNA non- treated (**top)** or LPS-treated groups (**middle**). Fold induction ratio (**bottom**). Statistical significance was determined using a two-way ANOVA with post hoc pairwise comparisons. ****$p < 0.0001$, **$p < 0.01$. *$p < 0.05$.

Antiviral	Antibiotics	Antimalarial	Antihelmintic
Bictegravir	Cephalexin	Artemether	Praziquantel
Boceprevir	Clindamycin	Primaquine	
Cobicistat	Erythromycin		
Dasabuvir	Flucloxacillin		
Delavirdine	Fusidic acid		
Indinavir	Midecamycin		
Nelfinavir	Telithromycin		
Paritaprevir	Ketoconazole		
Ritonavir	Cethromycin		
Telaprevir			
Tenofovir alafenamide			
Tipranavir			
Zidovudine			

Table 1 Supplemental

List of primer sequences

Primer	Forward	Reverse
BMAL1	GTGGCCTACTATCAGGCC	TGTCAATCATGTCTATACC
PER2	GTTTGTGTGGCCAACTCCAT	ACCAAACAGGACTGGGTCTC
CXCL10	GGTGAGAAGAGATGTCTGAATCC	GTCCATCCTTGGAAGCACTGCA
CXCL11	AAGGACAACGATGCCTAAATCCC	CAGATGCCCTTTTCCAGGACTTC
TNF-alpha	CTCTTCTGCCTGCTGCACTTTG	ATGGGCTACAGGCTTGTCACTC
IL-6	AGACAGCCACTCACCTCTTCAG	TTCTGCCAGTGCCTCTTTGCTG
IL-1 beta	CCACAGACCTTCCAGGAGAATG	GTGCAGTTCAGTGATCGTACAGG
ISG20	ACACGTCCACTGACAGGCTGTT	ATCTTCCACCGAGCTGTGTCCA
SLCO1B3_F	GTCACCTTGTCTAGCAGGATGC	GCATTCACCCAAGTGTGCTGAG
CYP3A4_F	CCGAGTGGATTTCCTTCAGCTG	TGCTCGTGGTTTCATAGCCAGC
CYP2B6	ACAGTGTGGAGAAGCACCGTGA	GGTTGAGGTTCTGGTGGCTGAA
gapdh	GTCTCCTCTGACTTCAACAGCG	ACCACCCTGTTGCTGTAGCCAA

Table 2 Supplemental
